# Complete sleep and local field potential analysis regarding estrus cycle, pregnancy, postpartum and post-weaning periods and homeostatic sleep regulation in female rats

**DOI:** 10.1038/s41598-020-64881-w

**Published:** 2020-05-22

**Authors:** Attila Tóth, Máté Pethő, Dóra Keserű, Dorina Simon, Tünde Hajnik, László Détári, Árpád Dobolyi

**Affiliations:** 1 In vivo Electrophysiology Research Group, Department of Physiology and Neurobiology, Eötvös Loránd University, Budapest, Hungary; 2 MTA-ELTE Laboratory of Molecular and Systems Neurobiology, Department of Physiology and Neurobiology, Hungarian Academy of Sciences and Eötvös Loránd University, Budapest, Hungary

**Keywords:** Neuroscience, Circadian rhythms and sleep, Motivation, Sexual behaviour, Social neuroscience

## Abstract

Sleep and local field potential (LFP) characteristics were addressed during the reproductive cycle in female rats using long-term (60–70 days) recordings. Changes in homeostatic sleep regulation was tested by sleep deprivation (SDep). The effect of mother-pup separation on sleep was also investigated during the postpartum (PP) period. First half of the pregnancy and early PP period showed increased wakefulness (W) and higher arousal indicated by elevated beta and gamma activity. Slow wave sleep (SWS) recovery was suppressed while REM sleep replacement was complete after SDep in the PP period. Pup separation decreased maternal W during early-, but increased during middle PP while did not affect during late PP. More W, less SWS, higher light phase beta activity but lower gamma activity was seen during the post-weaning estrus cycle compared to the virgin one. Maternal sleep can be governed by the fetuses/pups needs and their presence, which elevate W of mothers. Complete REM sleep- and incomplete SWS replacement after SDep in the PP period may reflect the necessity of maternal REM sleep for the offspring while SWS increase may compete with W essential for maternal care. Maternal experience may cause sleep and LFP changes in the post-weaning estrus cycle.

## Introduction

Changes in the brain associated with pregnancy (PREG) and lactation are being increasingly recognized. Consequently, our understanding of the brain regulatory mechanisms related to maternal care increased tremendously in recent years. Still, sleep disturbances remained frequent both during PREG^1,2^ and the postpartum (PP) period^3,4^ in mothers and may affect the development of the fetus^5^ as well as the emotional state of the mother^6^. Furthermore, the likelihood of PP depression correlates with the presence of sleep problems in the early PP period^7,8^. Since rodent models are particularly useful in understanding neuronal and hormonal changes in mothers, they can also be used to examine the mechanisms of maternal sleep changes. However, a comprehensive study on rodent sleep patterns during the reproductive cycle is missing. Therefore, the objective of the present study was to provide a detailed analysis about sleep and local field potential (LFP) changes in female rats to establish the basis for future studies in the field.

Previous studies found sleep changes during the different stages of the EST cycle^9–14^, PREG^13,15,16^ and the PP period^15,17,18^. However, most studies focused only on one given female stage and overlooked the fact that a continuum exists ranging from the nulliparous EST cycle to the primiparous post-weaning (PW) period with recurrent EST cycling. Assessment of this issue requires longitudinal *in vivo* electrophysiological studies but only one such study was performed to date^15^. Reported sleep-wake (S-W) changes during the EST cycle, PREG and the PP period indicated that homeostatic sleep drive may also change as a function of the actual female stage but this has not been studied using a longitudinal experimental design either. Only one study aimed to examine homeostatic sleep regulation by sleep deprivation (SDep) in female rats and that study focused on the EST cycle in virgins^9^. Homeostatic sleep regulation was not studied during either PREG or PP as previous studies using SDep or sleep restriction protocols during late PREG examined the effect of sleep loss on maternal behavior and endocrine profile^19^ or the effect of total rapid eye movements (REM) SDep on the maturation of S-W patterns of the offspring^20^. SDep during the PP period was used to block milk ejection and to increase suckling-induced prolactin (PRL) levels is dams previously separated from their pups^21^. In the present study, we performed long-lasting longitudinal *in vivo* local field potential (LFP) recordings ranging from the nulliparous EST cycle to the primiparous PW stage with recurring cycling in the same female rats. Our aim was to provide a detailed description of S-W and LFP patterns characteristic of nulliparous EST cycle, PREG, PP period and primiparous PW cycle. To also assess homeostatic sleep regulation, we performed SDep by 4-hours gentle handling during the virgin EST cycle, the first half of the PREG and different parts of the PP period.

Aside from the circadian^22^, homeostatic^23^ and neuronal regulation^24^, sleep is under endocrine control as well^25^. Levels of the gonadal hormones (e.g. PREG), their tropic- and releasing hormones show characteristic alternations during the estrus (EST) cycle^26–28^ as well as during different maternal stages^29^, Other hormones, such as PRL^30^ and oxytocin^31^ have elevated secretion during lactation. These profound hormonal changes may all contribute to adequate maternal behavior^30,32,33^. In addition, pups provide various somatosensory, olfactory, auditory and visual stimuli to rat dams^34–37^ to affect their mothers while rat dams provide various inputs to the fetuses and later to the pups to facilitate their development^38,39^. To address the effect of the presence of pups on the sleep and LFP of rat dams, 24-h mother-pup separation was performed at different parts of the PP period.

## Results

S-W and LFP findings are explained by consecutive maternal stages involving the whole reproductive cycle. The term “treatment” refers not only instrumental/concrete interventions (SDep, mother-pup separation) but to all periods which are considered to be different from the defined baselines regarding both their time point and physiological background. Key findings were summarized in Table 1.

**Table 1:** Summary of key findings.

maternal stage/treatment	sleep changes	LFP changes	SWS deprivation	REM deprivation	number of corrseponding figure(s)
virgin EST cycle	W ↓, SWS ↑ during MET LP W ↑, SWS ↓, REM ↓ during EST	delta, theta, beta, gamma ↑ during MET LP; theta ↓, alpha/sigma ↓, gamma ↑ during EST	strong rebound in sleep and strong delta power increase during recovery	overcompensation by the end of the day	2., S1 (sleep); 3. (LFP); 8., 9. (SD)
pregnancy 1st half	W ↑ (in LP); SWS ↓, REM ↓ (in LP)	delta, theta, alpha/sigma ↓, beta ↑ (in LP)	strong rebound in sleep and strong delta power increase during recovery	overcompensation by the end of the day	4., S2 (sleep); 5., S3 (LFP); 8., 9. (SD)
pregnancy 2nd half	REM ↓	beta ↑ (in LP), gamma ↑ (in DP)	n/a.	n/a.	4., S2 (sleep); 5., S3 (LFP)
early postpartum baseline	W ↑, SWS ↓ (in LP), REM ↓ (in LP)	delta, theta ↓; beta, gamma ↓ (in LP) delta, theta ↑ (in DP)	weak rebound and slight delta power increase during recovery	overcompensation by the end of the day	4., S2 (sleep); 5., S3 (LFP); 8., 9. (SD)
early postpartum pup separation	no change	no change	n/a.	n/a.	6., S4 (sleep); 7. (LFP)
middle postpartum baseline	W ↓, SWS ↑	delta ↑ (in DP); theta ↓, alpha/sigma ↓ (in LP)	no rebound in sleep, moderate increase in delta power during recovery	overcompensation by the end of the day	4., S2 (sleep); 5., S3 (LFP); 8., 9. (SD)
middle postpartum pup separation	W ↑ (in DP), SWS ↓ (in DP)	theta ↑; beta ↑ (in LP)	n/a.	n/a.	6., S4 (sleep); 7. (LFP)
late postpartum baseline	REM ↓ (in DP)	delta, theta, alpha/sigma ↓ (in LP); delta, theta ↑ (in DP);	moderate rebound in sleep, marginal increase in delta power during recovery	exact compensation by the end of the day	4., S2 (sleep); 5., S3 (LFP); 8., 9. (SD)
late postpartum pup separation	REM ↓ (in LP)	delta ↑, beta ↑ (in LP)	n/a.	n/a.	6., S4 (sleep); 7. (LFP)
post-weaning EST cycle	W ↑, SWS ↓, REM ↑ (during DIE1 LP); REM ↓ (during DIE2 DP)	delta, gamma ↓; beta ↑ (in LP)	n/a.	n/a.	2., S1 (sleep); 3. (LFP)

The table contains the major results in simplified format. See Results and Methods sections for details and appropriate baselines used for the comparisons. Symbols: ↑ - increase; ↓ - decrease. Abbreviations: EST – estrus; DP – dark phase; LFP – local field potential; LP – light phase; MET – metestrus; n/a – non-applicable; REM – rapid eye movements sleep; SWS – slow wave sleep; W – wakefulness.

### Estrus cycle – virgin

S-W and LFP changes were compared between the stages of the virgin EST cycle using diestrus 2nd day (DIE2) as baseline. Metestrus (MET) during the virgin EST cycle was characterized by higher amount of SWS (time X treatment, F (3, 72) = 11.14, p < 0.0001) and REM sleep (time X treatment, F (3, 72) = 16.3, p = 0.0092) and lower W (time X treatment, F (3, 72) = 24.2, p < 0.0001) in the light phase (LP) compared to the DIE2 baseline (Fig. 2A,B). DIE represented a stable stage regarding both sleep (Supplementary Fig. S1) and LFP parameters (Fig. 3) with only few significant deviations in different time points during DIE1 compared to DIE2 baseline (Supplementary Fig. S1A,B). However, these sporadic changes resulted in a significant decrease in W (time X treatment, F (3, 72) = 24.2, p = 0.0069) and significant increase in SWS (time X treatment, F (3, 72) = 11.14, p = 0.0176) during the dark phase (DP) of DIE1 (Fig. 2A,B).

**Figure 1:**
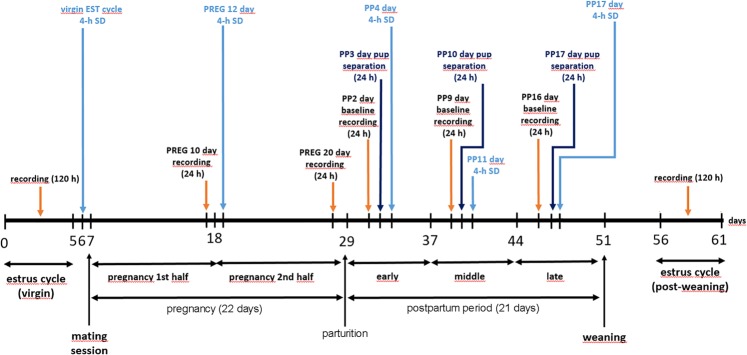
Experimental timeline for studying sleep and LFP changes during the virgin EST cycle, PREG, PP period and PW in female rats. The effects of mother-pup separation were also studied during the PP period. 4-h gentle handling sleep deprivation sessions were performed to study homeostatic sleep regulation during the virgin EST cycle, PREG and three consecutive thirds of the PP period. Note that the scheme shows an ideal case, some experiment lasted longer because mating session needed repetition. Furthermore, most but not all rats involved in the present study were subjected to all experiments depicted on the scheme.

**Figure 2:**
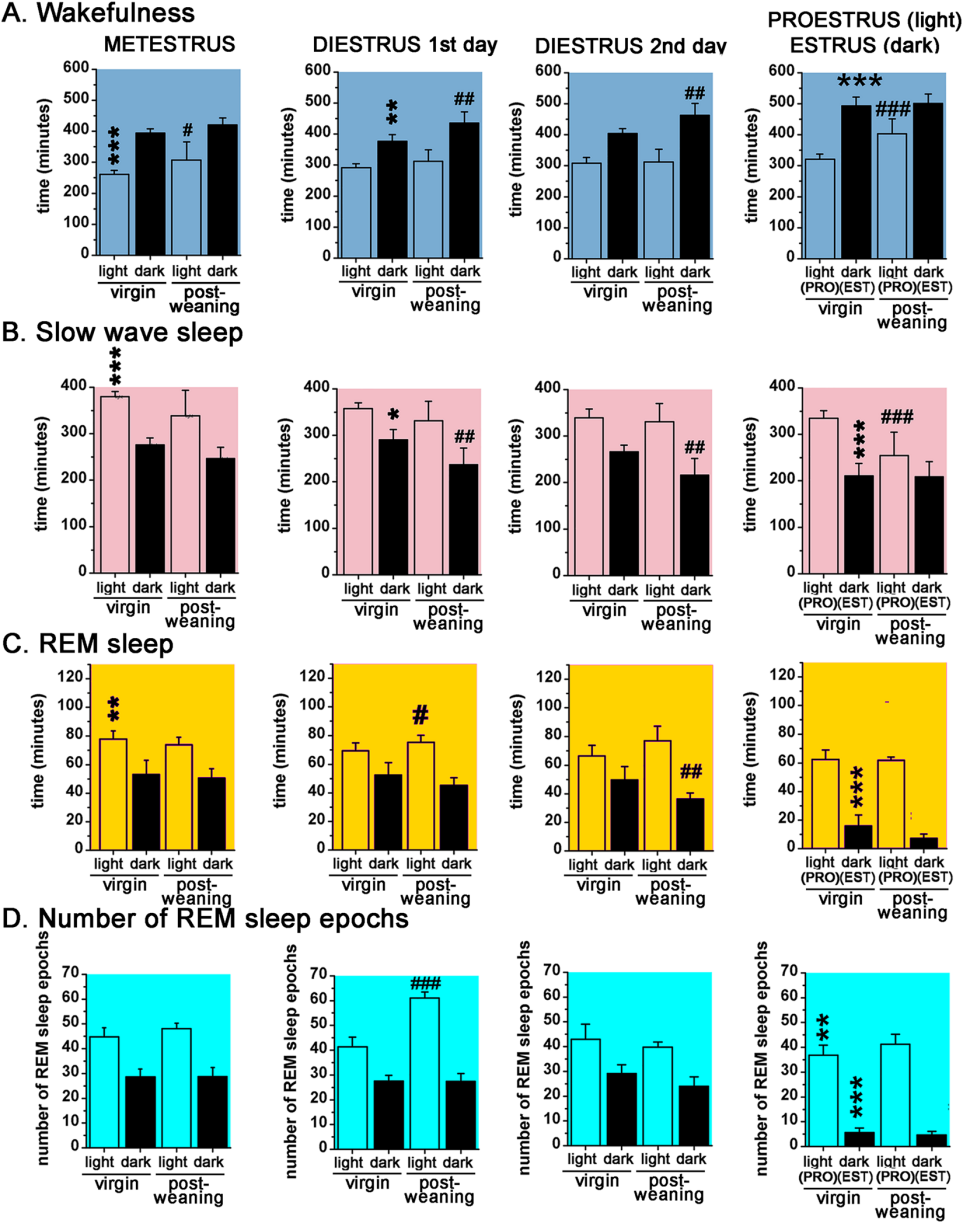
S-W amounts for the 12:12 hours LPs-DPs, respectively, during the virgin EST cycle (n = 10) and the PW EST cycle (n = 5), respectively (Panels A–C). Panel D shows the summarized number of REM sleep epochs for the 12:12 hours LPs-DPs, respectively. MET duration was taken to be 24 hours, DIE 48 hours (DIE 1st day and DIE 2nd day) while PRO and EST 12–12 hours, respectively. Black asterisks (*) indicate significant difference compared to the LP and DP values, respectively, of DIE2 day values as reference. Black hashes (**#**) indicate significant deviation of the PW light- and DP data, respectively, from corresponding virgin EST cycle data belonging to the same stage of the EST cycle. Significance was tested with two-way ANOVA with time and treatment as factors, followed by Sidak’s multiple comparisons test. Significance levels: *, # - p < 0.05; **, ##- p < 0.01; ***, ### - p < 0.001. Data are expressed as mean ± S.E.M.

W was significantly enhanced (time X treatment, F (3, 72) = 24.82, p < 0.0001) while SWS was significantly attenuated (time X treatment, F (3, 72) = 11.14, p < 0.0001) during the EST night in line with the reduction of REM sleep time (Fig. 2A,B). During both EST cycles, the most prominent S-W change was the dramatic decrease of REM sleep during the EST night, which started at the end of the proestrus (PRO) stage (Supplementary Fig. S1C). The decrease was 67.9% compared to DIE2 baseline (time X treatment, F (3, 72) = 16.3, p < 0.0001) (Fig. 2C). Decrease of the number of REM sleep epochs was the cause of the strong drop of REM sleep time (80.2% decrease compared to DIE2 baseline; time X treatment, F (3, 72) = 24.82, p < 0.0001) (Supplementary Fig. S1D).

**Figure 3:**
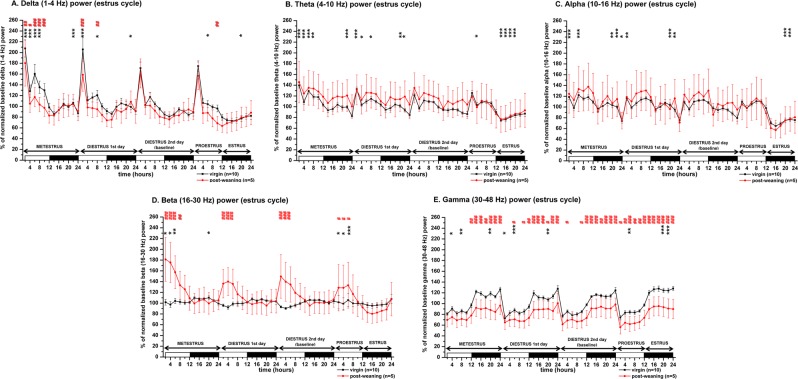
LFP power changes during the virgin EST cycle (n = 10, thick black line) and the PW EST cycle (n = 5, thin red line). Panel A: delta power (1–4 Hz); panel B: theta power (4–10 Hz); panel C: alpha/sigma power (10–16 Hz); panel D: beta power (16–30 Hz); panel E: gamma power (30–48 Hz). Power values are expressed as percentage of normalized baseline (DIE2 day of the virgin EST cycle) values and summarized in 2-h long blocks. MET duration was taken to be 24 hours, DIE 48 hours (DIE 1st day and DIE 2nd day) while PRO and EST 12–12 hours, respectively. White and black bars at the X axis represent light- and DPs, respectively. Black asterisks (*) indicate significant deviation from the corresponding baseline value in case of the virgin EST cycle data. DIE2 day values were defined as baseline. Red hashes (#) indicate significant deviation in a point-to-point comparison from the corresponding reference value in case of the PW EST cycle data. The corresponding virgin EST cycle data were defined here as reference. Significance was tested with two-way ANOVA with time and treatment as factors, followed by Sidak’s multiple comparisons test. Significance levels: *,# - p < 0.05; **,## - p < 0.01; ***,### - p < 0.001. Data are expressed as mean ± S.E.M.

According to the increased amount of SWS during MET LP, normalized delta (1–4 Hz) power expressed as baseline % significantly increased (time X treatment, F (33, 432) = 11.05, p < 0.001) (Fig. 3A). There was a decreasing trend in maximal delta power seen at the beginning of the LP from MET to PRO (Fig. 3A) reflecting decreasing homeostatic sleep drive in the LP towards the active stages (PRO and EST) of the cycle.

According to the strong decrease of the REM sleep during EST (Fig. 2C), theta (4–10 Hz) power was also significantly reduced during EST compared to the DIE2 baseline (time X treatment, F (33, 432) = 18.05, p < 0.001) (Fig. 3B). Alpha/sigma power was also significantly reduced during the EST night (time X treatment, F (33, 432) = 30.79, p < 0.001) (Fig. 3C). LP during the MET stage of the EST cycle generally showed an increase in the power of slower waves including theta compared to DIE2 baseline (Fig. 3B). Interestingly, in the range of faster beta (Fig. 3D) and gamma (Fig. 3E) activities, increases were found too (time X treatment, F (33, 432) = 7.991, p < 0.001 for beta power; time X treatment, F (33, 432) = 12.36, p < 0.001 for gamma power).

Beta power showed mild circadian variation with higher values during the DP (Fig. 3D). Across the cycle, the highest beta power was found during PRO LP and MET LP compared to DIE2 LP values representing local minima and baseline at the same time.

Gamma power showed strong circadian fluctuation as higher power values were seen during the DP according to the higher level of W (Fig. 3E) while lower values characterized the LP. Gamma power was similar during MET, DIE1 and DIE2 nights but showed significant elevation during the EST night (time X treatment, F (33, 432) = 12.36, p < 0.001) (Fig. 3E).

### Pregnancy

**Figure 4:**
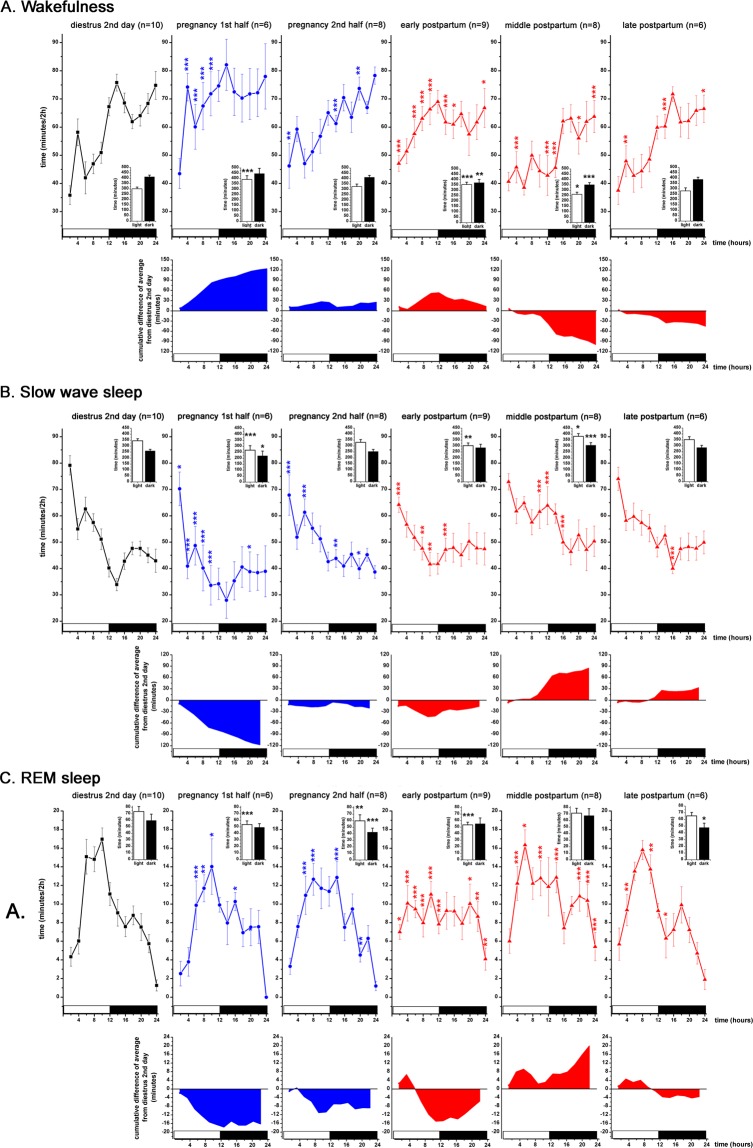
S-W changes during the DIE2 day of the virgin EST cycle (n = 10, black line), during the first half of the PREG (PREG10 day, n = 6, blue line), second half of the PREG (PREG20 day, n = 8, blue line), early PP (PP2 day, n = 9, red line), middle PP (PP9 day, n = 8, red line) and late PP (PP16 day, n = 6, red line). Panel A: W; panel B: SWS; panel C: REM sleep. On each panel, cumulative difference of the averages from the DIE2 day (baseline) average are shown to help assess the direction of changes seen during PREG and PP period, respectively, compared to DIE2 baseline. Data were analyzed in 2-h long bins and expressed as minutes/2-h. Small insets show summarized W (panel A), SWS (panel B) and REM sleep (panel C) amounts for the 12:12 hours light-DPs, respectively (Panel A-C). White and black bars at the X axis represent light- and DPs, respectively. Blue asterisks (*) indicate significant difference from the DIE2 baseline during PREG10 and PREG20 days, respectively. Red asterisks (*) indicate significant deviation during PP2, PP9 and PP19 days, respectively, compared to DIE2 baseline. In the insets, black asterisks (*) indicate significant difference from the LP and DP DIE2 values values, respectively. Significance was tested with two-way ANOVA with time and treatment as factors, followed by Sidak’s multiple comparisons test. Significance levels: *,*,* - p < 0.05; **,**,** - p < 0.01; ***,***,*** - p < 0.001. Data are expressed as mean ± S.E.M.

In the first half of the PREG (PREG10 day), more W was present during the LP (Fig. 4A) compared to the DIE2 baseline (time X treatment, F (5, 82) = 7.538, p < 0.0001) as the number of W epochs increased significantly (Supplementary Fig. S2A) (time X treatment, F (5, 82) = 5.428, p = 0.0051). SWS was significantly decreased during both the LP and the DP compared to DIE2 baseline (time X treatment, F (5, 82) = 3.949; p < 0.0001 for the light, p < 0.05 for the dark) with stronger reduction during the LP. Overall, SWS values were shifted downwards during the whole day resulting in ∼120 minutes of sleep decrement at the end of the DP compared to DIE2 baseline as seen by the cumulative difference of averages (Fig. 4A, 2nd row). W increase appeared at the expense of the SWS decrease as cumulative increase of W (Fig. 4A, 2nd row) was similar to the cumulative decrease of SWS. REM sleep also decreased during the LP (Fig. 4C) (time X treatment, F (5, 82) = 4.222, p < 0.0001).

**Figure 5:**
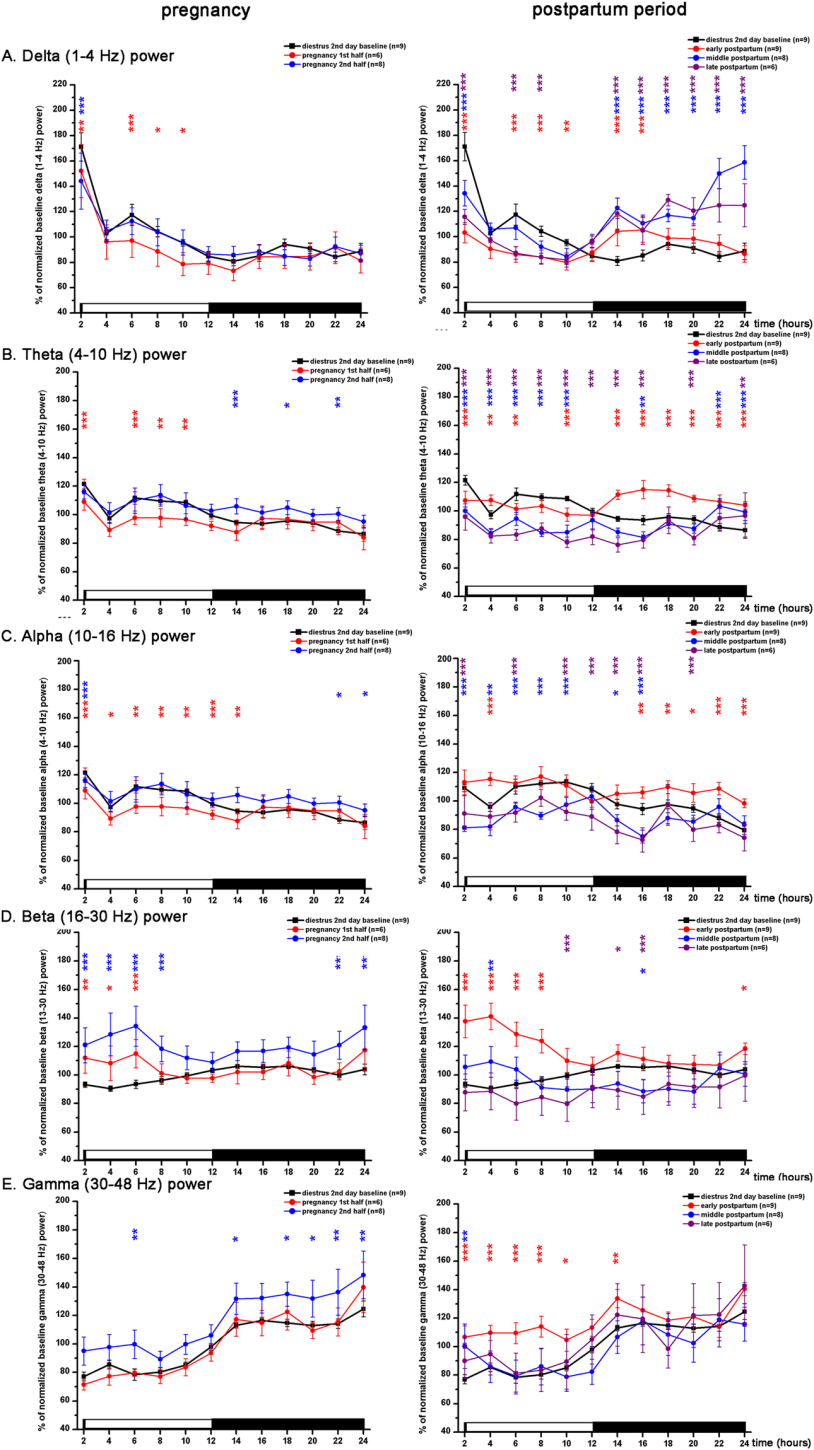
LFP power changes during the PREG and postpartum periods. LFP is shown for DIE2 day of the virgin EST cycle (n = 10), the first half of the PREG (PREG10 day, n = 6), the second half of the PREG (PREG20 day, n = 8), early PP (PP2 day, n = 9), middle PP (PP9 day) and late PP (PP16 day, n = 6). Panel A: delta power (1–4 Hz); panel B: theta power (4–10 Hz); panel C: alpha/sigma power (10–16 Hz); panel D: beta power (16–30 Hz); panel E: gamma power (30–48 Hz). Power values are expressed as percentage of normalized baseline (DIE2 day of the virgin EST cycle) values and summarized in 2-h long blocks. Red asterisks (*) indicate significant deviation during PREG10 day (left column) or PP2 day (right column), respectively, compared to DIE2 baseline. Blue asterisks (*) indicate significant deviation during PREG20 day (left column) or PP9 day (right column), respectively, compared to DIE2 baseline. Purple asterisks (*) indicate significant deviation during PP16 day (right column), respectively, compared to DIE2 baseline. Significance was tested with two-way ANOVA with time and treatment as factors, followed by Sidak’s multiple comparisons test. Significance levels: *,*,* - p < 0.05; **,**,** - p < 0.01; ***,***,*** - p < 0.001. Data are expressed as mean ± S.E.M.

During the second half of the PREG (PREG20 day), W increased significantly only in some points compared to DIE2 baseline values and no difference was seen in the amount of W summarized to the light- and to the DP (Fig. 4A). Interestingly, length of W epochs significantly increased in the DP (Supplementary Fig. S2B) (time X treatment, F (5, 82) = 2.772, p = 0.0109) but this was counterbalanced by the decrease of the number of W epochs (Fig. 4A) (time X treatment, F (5, 82) = 5.428, p = 0.0044). Complementing the W increase in separate time points, SWS was significantly reduced during the same time points (Fig. 4B). REM sleep was significantly reduced during the LP (Fig. 4C) (time X treatment, F (5, 82) = 4.222, p = 0.0016) as well as during the DP (time X treatment, F (5, 82) = 4.222, p < 0.0001). According to the reduced amount of SWS during the LP (Fig. 4B), delta power was also decreased (Fig. 5A) together with the theta- as well as alpha/sigma power (Fig. 5B,C) while in the region of the faster rhythms, increase was seen in the beta power (Fig. 5D).

Increased W during the LP in PREG10 day was accompanied by decreased power in the range of slower LFP activities (delta, theta, alpha/sigma) (Fig. 5A–C) and increase in the beta range (Fig. 5D). LFP power analysis by vigilance levels showed that general increase of beta power compared to DIE2 baseline was due to the increase during W (Supplementary Fig. S3A).

Beta (13–30 Hz) power was generally elevated during the whole day on PREG20 day (time X treatment, F (11, 180) = 10.12, p < 0.001) (Fig. 5D). This was caused primarily by the increase of beta power during W during most of the day (time X treatment, F (11, 180) = 11.72, p < 0.001) (Supplementary Fig. S3A) and by a lesser extent, beta increment during SWS (time X treatment, F (11, 180) = 2.467, p = 0.007; Supplementary Fig. S3B). Similar to the EST cycles (Fig. 3E), gamma (30–48 Hz) power showed clear circadian variation during PREG too with higher values during the DP (Fig. 5E). Power analysis by vigilance levels showed that most of the gamma power resided in W compared to SWS and REM sleep during DIE2 baseline (Supplementary Fig. S3D–F). Overall gamma power increment observed during whole day at PREG20 was derived mainly from the increase during W (time X treatment, F (11, 180) = 2.601, p = 0.004; Supplementary Fig. S3D). Gamma power increase during SWS had less importance even if the difference compared to DIE2 baseline was larger in case of SWS compared to W (see Fig. Supplementary Fig. S3E vs. S3D).

### Postpartum baseline

Early postpartum (PP2 day) showed the strongest S-W changes among the three parts (represented by the PP2, PP9 and PP16 days) of the PP period compared to DIE2 baseline. PP2 day was characterized by elevated W during the LP while W was below the baseline during the DP (time X treatment, F (5, 82) = 7.538; p = 0.0001 for the LP, p = 0.0084 for the DP; Fig. 4A). Decreased W in the DP originated from the drop of both the number of epochs (Supplementary Fig. S2A) (time X treatment, F (5, 82) = 5.428; p < 0.0001) and the length of the epochs (Supplementary Fig. S2B) (time X treatment, F (5, 82) = 2.772; p = 0.0076). Increase of W during the LP appeared at the expense of the reduced SWS (Fig. 4B) (time X treatment, F (5, 82) = 3.949; p = 0.0034) and REM sleep (Fig. 4C) (time X treatment, F (5, 82) = 4.222; p < 0.0001). REM sleep decrease was caused by the drop of epoch number during the LP (Supplementary Fig. S2A) (time X treatment, F (5, 82) = 6.016; p = 0.0008). Circadian variation disappeared in case of all three vigilance stages as nearly equal amount of W, SWS and REM sleep was present, respectively, during the light- and the DPs (Fig. 4A–C insets).

During middle PP (PP9 day), W significantly decreased in both phases (time X treatment, F (5, 82) = 7.538; p = 0.0164 for the LP, p = <0.0001 for the DP) compared to DIE2 baseline (Fig. 4A). Number of W epochs decreased in both phases (Supplementary Fig. S2A) (time X treatment, F (5, 82) = 5.428; p = 0.0020 for the LP, p < 0.0001 for the DP). At the end of the day, cumulative decrease of W exceeded 99 minutes compared to DIE2 baseline (Fig. 4A, 2nd row). SWS was significantly increased in both phases (Fig. 4B) (time X treatment, F (5, 82) = 3.949; p = 0.0183 for the LP, p = 0.0009 for the DP) and cumulative SWS difference was 84.59 minutes at the end of the day. REM sleep was significantly elevated in several time points during both phases but the change was not significant for neither of the 12-h phases (Fig. 4C inset).

Late PP (PP16 day) showed few sleep differences compared to DIE2 baseline. Only REM sleep in the DP showed a moderate decrease (time X treatment, F (5, 82) = 4.222; p = 0.0182) (Fig. 4C inset).

Increased W and decreased sleep during PP2 day was accompanied by decreased delta- (Fig. 5A), theta- (Fig. 5B) and increased beta- (Fig. 5D) and gamma (Fig. 5E) power during the LP. In the DP, delta-, theta- and alpha/sigma power (Fig. 5C) were increased. Power analysis by vigilance stages showed a very strong increase of beta power during W (time X treatment, F (11, 192) = 37.16; p < 0.001) (Supplementary Fig. S3A) during the LP while SWS beta power was elevated during all day (time X treatment, F (11, 192) = 7.202; p < 0.001) (Supplementary Fig. S3B). Strong elevation of beta power during REM sleep in the first third of the LP (time X treatment, F (11, 192) = 31.22; p < 0.001) (Supplementary Fig. S3C) also contributed in the general beta power elevation seen in this period (time X treatment, F (11, 192) = 41.1; p < 0.001) (Fig. 5D). Gamma power increase during W (time X treatment, F (11, 192) = 15.07; p < 0.001) (Supplementary Fig. S3D) was the main source of the general gamma power increase seen during the LP (time X treatment, F (11, 192) = 18.89; p < 0.001) (Fig. 5E) but REM sleep gamma power increase also contributed in the gamma elevation seen during the first third of the LP (Fig. 5E).

Elevation of night sleep seen during PP9 day was accompanied by increased delta power during the DP (time X treatment, F (11, 156) = 84.5; p < 0.001) (Fig. 5A). Suppressed W was reflected by the decrease of theta power throughout the day (time X treatment, F (11, 156) = 48.08; p < 0.001) (Fig. 5B). Overall beta power showed only sporadic significant changes in two time points (Fig. 5D) but power analysis by vigilance levels indicated significant W beta power decrease mainly during the DP (time X treatment, F (11, 180) = 21.35; p < 0.001) (Supplementary Fig. S3A).

No SWS change was observed during PP16 day compared to DIE2 baseline. However, delta power was significantly decreased during the LP (Fig. 5A) while significant increase was seen during the whole DP. Theta- and alpha/sigma power was generally depressed during the whole day compared to DIE2 baseline (time X treatment, F (11, 180) = 22.09; p < 0.001 for the theta power; time X treatment, F (11, 156) = 5.25; p < 0.001 for the alpha/sigma power; Fig. 5B,C).

### Postpartum treatment - mother-pup separation

Mother-pup separation sessions, performed on PP3, PP10 and PP17 days, lasted for 24 h.

**Figure 6:**
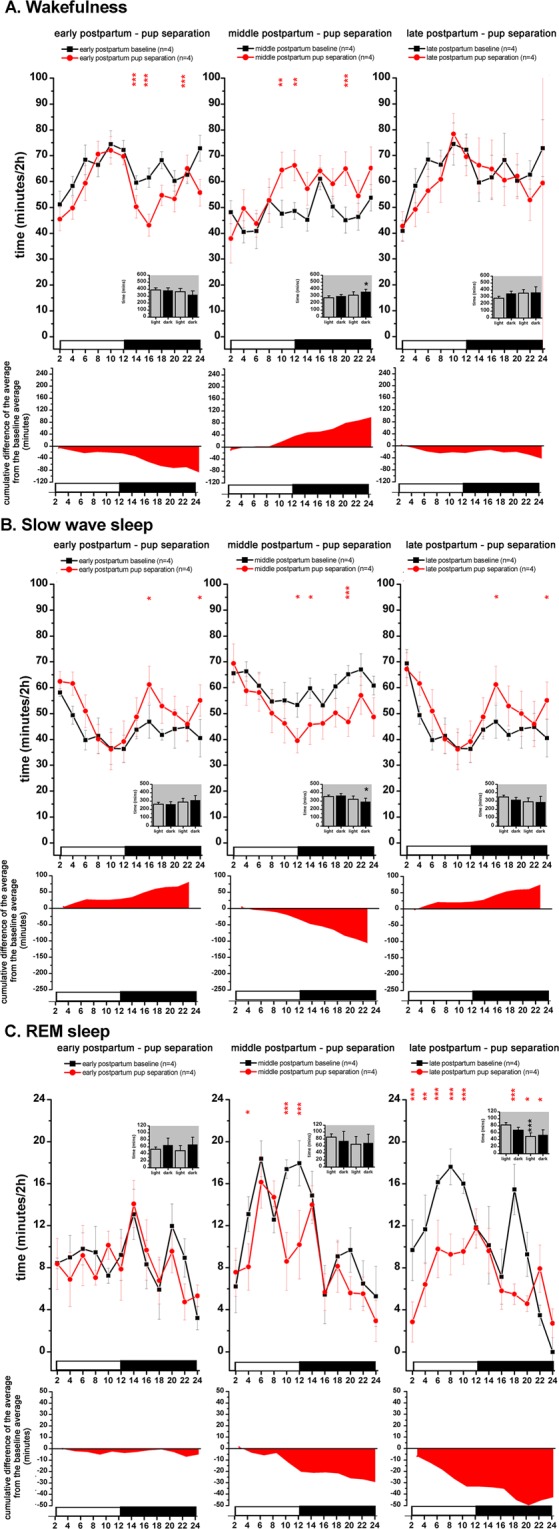
S-W changes during the mother-pup separation sessions. From left to right: separation sessions performed during early PP (n = 4, PP3 day), middle PP (n = 4, PP10 day) and late PP (n = 4, PP17 day). Corresponding PP baseline recordings served as baseline (n = 4, PP2 for PP3 separation; n = 4, PP9 for PP10 separation; n = 4, PP16 for PP17 separation; n = 5). Averaged baseline values were calculated from the identical rats subjected to the actual treatment in each case. Panel A: W; panel B: SWS; panel c: REM sleep. Black lines indicate baseline values, red lines show pup separation values. On each panel, cumulative difference of the averages from the corresponding baseline average is depicted helping to assess the direction of the changes as well as the accumulating S-W deficit or excess regarding homeostatic sleep regulation. Data were analyzed in 2-h long bins and expressed as minutes/2-h. Small insets show summarized W (panel A), SWS (panel B) and REM sleep (panel C) amounts for the 12:12 hours light-DPs, respectively (Panel A-C). White and black bars at the X axis represent light- and DPs, respectively. Red asterisks (*) indicate significant deviation from the corresponding baseline value. In the insets, black asterisks (*) indicate significant difference of the LP and DP values, respectively, compared to the corresponding baseline. Significance was tested with two-way ANOVA with time and treatment as factors, followed by Sidak’s multiple comparisons test. Significance levels: *,* - p < 0.05; **,** - p < 0.01; ***,*** - p < 0.001. Data are expressed as mean ± S.E.M.

**Figure 7:**
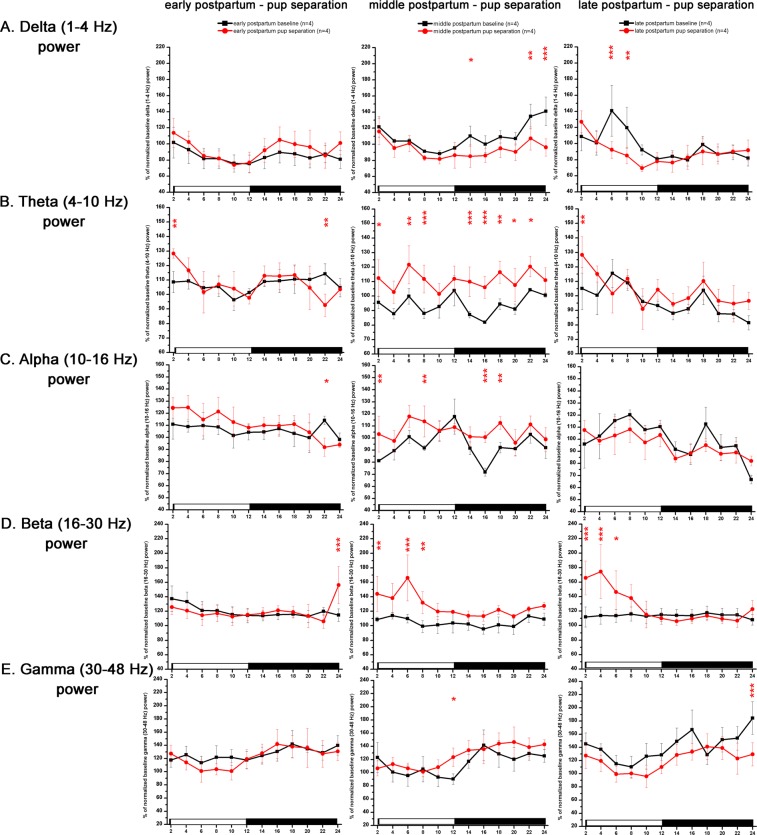
LFP power changes during the mother-pup separation sessions. From left to right: separation sessions performed during early PP (n = 4, PP3 day), middle PP (n = 4, PP10 day) and late PP (n = 4, PP17 day). Corresponding PP baseline recordings served as baseline (n = 4, PP2 for PP3 separation; n = 4, PP9 for PP10 separation; n = 4, PP16 for PP17 separation; n = 5). Panel A: delta power (1–4 Hz); panel B: theta power (4–10 Hz); panel C: alpha/sigma power (10–16 Hz); panel D: beta power (16–30 Hz); panel E: gamma power (30–48 Hz). Power values are expressed as % of normalized corresponding baseline values and summarized in 2-h long blocks. Averaged baseline values were calculated from the identical rats subjected to the actual treatment in each cases. White and black bars at the X axis represent light- and DPs, respectively. Red asterisks (*) indicate significant deviation from the corresponding baseline value. Significance was tested with two-way ANOVA with time and treatment as factors, followed by Sidak’s multiple comparisons test. Significance levels: * - p < 0.05; ** - p < 0.01; *** - p < 0.001. Data are expressed as mean ± S.E.M.

Pup separation on PP3 day did not cause significant sleep and LFP changes in the LP (Figs. 6A and 7A–E). During the DP, W decreased significantly in 3 time points (6 hours altogether) because of the decrease in the number of W epochs (Supplementary Fig. S4A) (treatment, F (1, 12) = 8.806; p = 0.0358) while SWS showed significant elevation in two time points (Fig. 6B). Taken together the whole day, W was below the baseline during most of the day while SWS was moderately elevated but did not reach statistical significance except for two time points during the DP (Fig. 6B). The cumulative deficit in the W and the resulting increment for SWS at the end of the day was 87.1 and 81.0 minutes, respectively.

S-W dynamics was the opposite during the PP10 day pup separation compared to the PP3 day one. W was increased during the second half of the LP and during the DP (treatment, F (1, 12) = 8.21; p = 0.0482) while decrease was seen in SWS in the same time periods (DP: treatment, F (1, 12) = 11.13; p = 0.0147). REM sleep was also below the baseline level in 3 time points during the LP (Fig. 6C) as REM sleep epoch length was significantly decreased in the LP (Supplementary Fig. S4B) (treatment, F (1, 12) = 27.17; p = 0.0060) compared to baseline. Length of REM sleep epochs was also reduced in the DP (treatment, F (1, 12) = 27.17; P = 0.0065).

Pup separation during late PP (performed on PP17 day) did not caused change in the W of the dams (Fig. 6A, third column). SWS during the DP tended to be elevated but the increase was only significant in two distant time points while cumulative increase in SWS was 74.5 minutes compared to baseline (Fig. 6B, third column). REM sleep was strongly affected by pup separation (Fig. 6C, third column). REM sleep was below of the baseline level during most of the day, the effect decrease was stronger in the LP (treatment, F (1, 12) = 21.78; p = 0.0009). Shortening of REM sleep epochs both during the light- and the DPs (Supplementary Fig. S4B) (treatment, F (1, 14) = 28.51; p = 0.0004 for the LP, p = 0.0498 for the DP) was accounted for the decrease of REM sleep time. Generally, effect of pup isolation on REM sleep became stronger as a function of time spent in the PP period as seen on Fig. 6C from left to right.

Pup separation caused only negligible LFP changes on PP3 day (Fig. 7, left column). On PP10 day, pup separation was associated with increased theta power during most of the day (treatment, F (11, 72) = 5.103; p = <0.001) (Fig. 7B) and increased beta power during the LP (treatment, F (11, 72) = 5.38; p = <0.001) (Fig. 7D) as a consequence of beta power increase seen during SWS (data not shown). Elevated W theta power during the whole day and increased SWS theta power during the LP caused the overall theta power elevation on PP10 day (data not shown).

### Total sleep deprivation - effect on SWS

**Figure 8:**
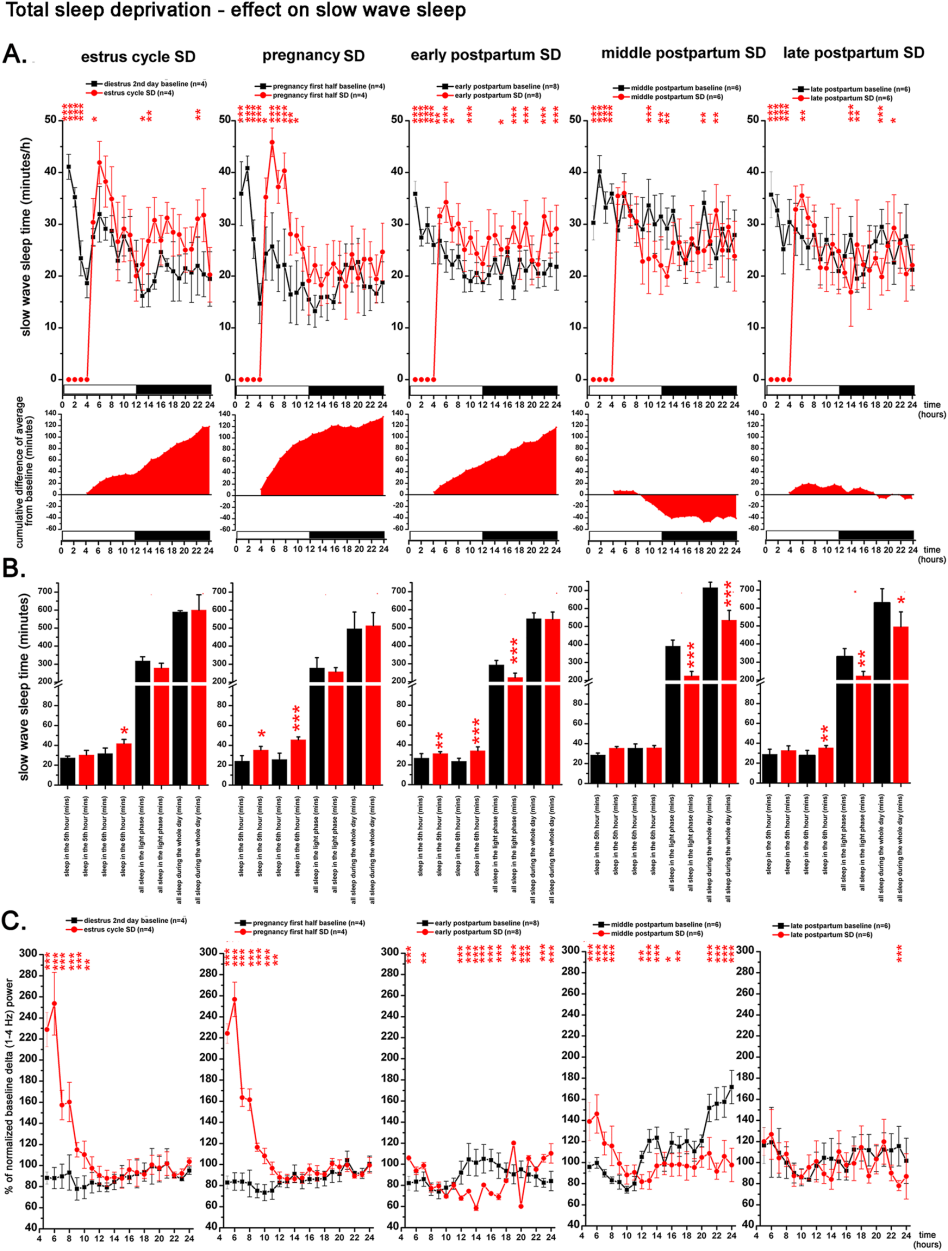
Effect of 4-h gentle handling total sleep deprivation on SWS and delta power during the virgin EST cycle (n = 4), PREG (n = 4, PREG12 day), early PP period (n = 8, PP4 day), middle PP period (n = 6, PP11 day), and late PP period (n = 6, PP17 day). Corresponding baselines in the same order: DIE2 day of the virgin EST cycle (n = 4), PREG10 day (n = 4), PP2 (n = 8), PP9 (n = 6) and PP16 (n = 6) days. Panel A: SWS times in minutes/h. Black lines indicate baseline values, red lines show values after SDep. Cumulative difference of the averages from the corresponding baseline shows the dynamics of SWS compensation in time after SDep. Panel B: SWS times in minutes summarized in 4 different periods (5^th^ hour; 6^th^ hour; whole LP; whole day) after the 4-h SDep session. Black columns: baseline values, red columns: values after SDep. Panel C: changes of normalized delta (1–4 Hz) power expressed as percentage of corresponding baseline. Black lines: baseline; red lines: after SDep. Note that SDep caused complete SWS suppression in the deprivation period (panel A). Also note that delta power values belonging to the deprivation period as well as to the corresponding baseline period are not plotted on panel C. Averaged baseline values were calculated from the identical rats subjected to the actual SDep session in each cases. White and black bars at the X axis represent light- and DPs, respectively. On panel A and C, red asterisks (*) indicate significant deviation from the corresponding baseline value. Significance was tested by two-way ANOVA with time and treatment as factors, followed by Sidak’s multiple comparisons test. On panel B, SWS times in the 5th and 6th hours after SDep, respectively, were compared to corresponding baseline values as parts of time series using two-way ANOVA. SWS times during the whole LP and during the whole day in case of SDep were compared with the corresponding baseline values using Welch’s t-test. Significance levels: * - p < 0.05; ** - p < 0.01; *** - p < 0.001. Data are expressed as mean ± S.E.M.

During the SDep sessions, SDep was achieved as no sleep occurred in any of the rats subjected to the 4-h gentle handling SDep in any of the sessions. Intensity of rebound sleep was characterized by delta power enhancement after the SDep when sleep was allowed again. For this reason, delta power values were analyzed hourly and power values seen during the SDep period as well as during the corresponding baseline recordings were not plotted on Figs. 8. and 9.

Strong and similar sleep rebound (Fig. 8A) and delta power increase (Fig. 8B) appeared in case of virgin EST cycle SDep as well as in case of the SDep performed during PREG12 day. Interestingly, the strongest increase in SWS and delta power was seen in the 6th hour and not immediately after the deprivation period (in the 5th hour). Delta power was more than threefold in the 6th hour compared to corresponding baseline (Fig. 8C) and delta power remained significantly elevated until the 11th hour in case of the EST cycle SDep and until the 12th hour after the PREG12 day SDep. All lost sleep was replenished until the end of the LP and overall SWS amount during the whole day did not differ compared to the corresponding baseline.

In case of early PP SDep performed on PP4 day, only a weak rebound appeared in the 5th and 6th hours and significant SWS loss was seen at the end of the LP (Welch’s t-test, p < 0.0001). Although the pace of the sleep compensation was slow, lost sleep was completely recovered by the end of the day. Sleep compensation was accompanied by low delta power except for the first 3 hours after the SDep, delta power remained below the baseline level during most of the day.

After the middle PP SDep session performed on PP11 day, no rebound was seen as SWS time showed negligible elevation in the 5th and 6th hours (Fig. 8A,B). SWS times were lower in the second half of the LP compared to baseline and sleep recovery was not completed until the end of the LP (Welch’s t-test, p < 0.0001). Significant sleep debt was seen even at the end of the DP (Welch’s t-test, p = 0.0001). On the level of delta activity, moderate rebound was seen between the 5th and 10 h hours of the LP (Fig. 8C), but delta power remained below the baseline level in the following period (hours 11–24).

Late PP SDep on PP17 day was followed by moderate SWS rebound in the 6th hour (Fig. 8A,B) with minimal delta power increase (Fig. 8C). Complete sleep compensation did not appear either at the end of the LP (Welch’s t-test, p = 0.0012) or at the end of the day (Welch’s t-test, p = 0.0185). Delta power increase was completely absent after the SDep period (Fig. 8C, left).

### Total sleep deprivation - effect on REM sleep

**Figure 9:**
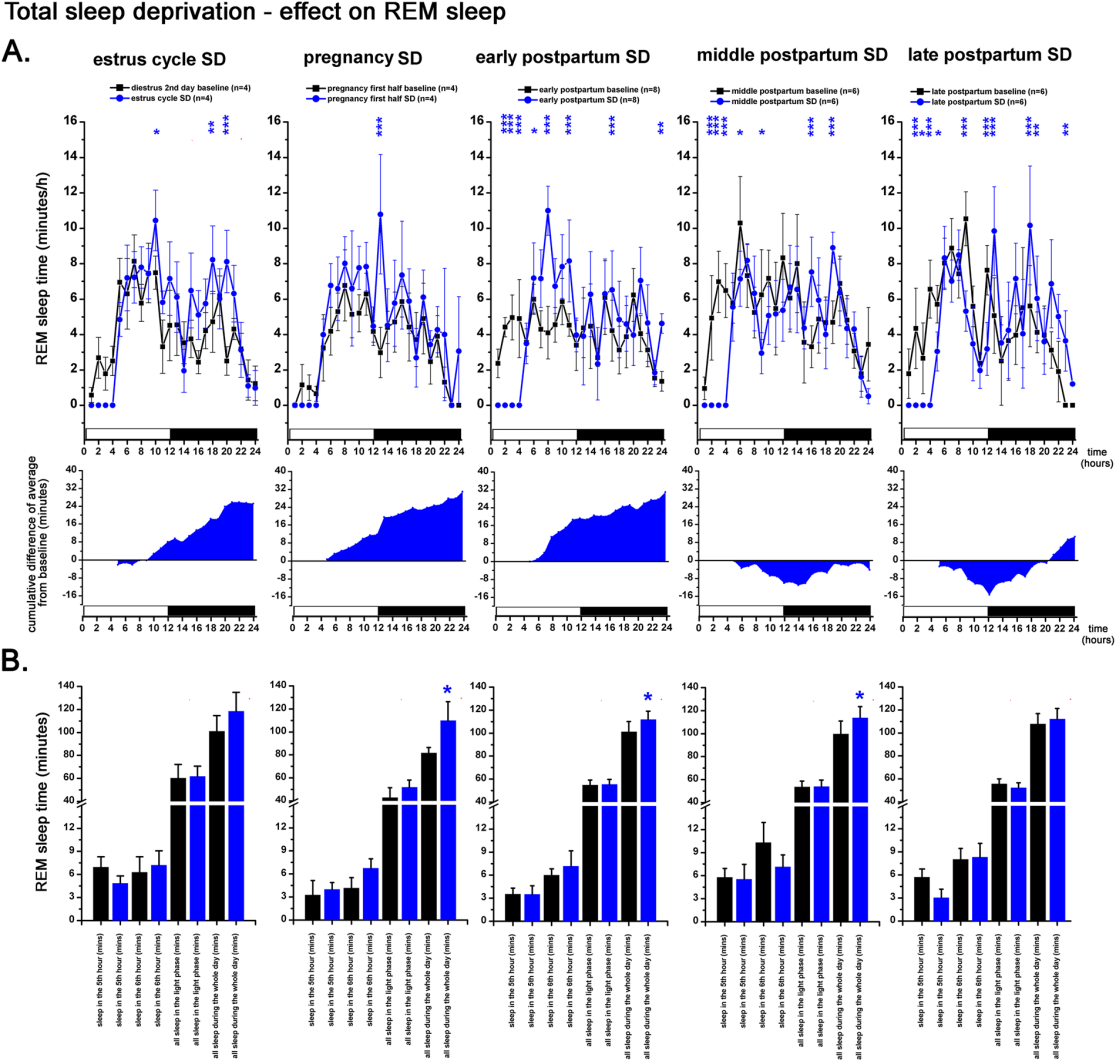
Effect of 4-h gentle handling total sleep deprivation on REM sleep during the virgin EST cycle (n = 4), PREG (n = 4, PREG12 day), early PP period (n = 8, PP4 day), middle PP period (n = 6, PP11 day), late PP period (n = 6, PP17 day). Corresponding baselines in the same order: DIE2 day of the virgin EST cycle (n = 4), PREG10 day (n = 4), PP2 (n = 8), PP9 (n = 6) and PP16 (n = 6) days. Panel A: REM sleep times in minutes/h. Black lines indicate baseline values, blue lines show values after SDep. Cumulative difference of the averages from the corresponding baseline shows the dynamics of REM sleep compensation in time after SDep. Panel B: REM sleep times in minutes summarized in 4 different periods (5^th^ hour; 6^th^ hour; whole LP; whole day) after the 4-h SDep session. Black columns: baseline values, blue columns: values after SDep. Note that SDep caused complete REM sleep suppression in the deprivation period (panel A). Averaged baseline values were calculated from the identical rats subjected to the actual SDep session in each case. White and black bars at the X axis represent light- and DPs, respectively. On panel A and C, blue asterisks (*) indicate significant deviation from the corresponding baseline value. Significance was tested by two-way ANOVA with time and treatment as factors, followed by Sidak’s multiple comparisons test. On panel B, REM sleep times in the 5th and 6th hours after SDep, respectively, were compared to corresponding baseline values as parts of time series using two-way ANOVA. REM sleep times during the whole LP and during the whole day in case of SDep were compared with the corresponding baseline values using Welch’s t-test. Significance levels: * - p < 0.05; ** - p < 0.01; *** - p < 0.001. Data are expressed as mean ± S.E.M.

REM sleep loss was significantly overcompensated by the end of the day in case of SDeps performed during PREG on PREG12 day (Welch’s t-test, p = 0.0373), early PP on PP4 day (Welch’s t-test, p = 0.0225) and middle PP on PP11 day (Welch’s t-test, p = 0.0445) (Fig. 9A,B). In case of late PP SDep performed on PP16 day, REM sleep loss was compensated to the same level of REM sleep seen during the corresponding baseline period (PP16 day) (Fig. 9B, right).

### Estrus cycle - post-weaning

Another 120-h long recording was made starting after 5 days of weaning to assess one EST cycle after maternal experience was gained. To assess any potential difference between virgin vs. PW cycles, point-to-point comparisons were made.

W and SWS values were generally higher and lower, respectively, during the PW cycle compared to the virgin one (Supplementary Fig. S1A and S1B), but temporal dynamics of all three S-W stages were the same during both cycles despite the amplitude differences. During the PW cycle, MET had significantly more W during the LP (time X treatment, F (7, 104) = 3.543, p = 0.0206) compared to the virgin EST cycle (Fig. 2A).

In the DIE of the PW cycle, W was shifted towards the higher values (time X treatment, F (7, 104) = 3.543, p = 0.0011 for both DIE1 and DIE2 nights) while SWS was shifted towards lower levels during both DIE nights (time X treatment, F (7, 104) = 3.025, p = 0.0029 for DIE1 night, p = 0.0076 for DIE2 night) compared to the virgin EST cycle (Supplementary Fig. S1A and S1B). Lower amount of SWS during PW DIE2 night was accompanied by significantly lower REM sleep time (time, F (7, 104) = 5.584, p = 0.0048) compared to virgin cycle values as baseline.

The amount of REM sleep during the EST night was decreased further during the PW cycle, but the difference was not significant in the virgin vs. PW cycle comparison (Fig. 2C). REM sleep reduction was caused by the strong decrease of the REM sleep epoch number (Fig. 2D) as it was seen during the virgin EST cycle. Enhanced W and attenuated SWS were observed during the EST night in case of the PW cycle too, but no further changes were seen in the parameters compared to the virgin EST cycle (Fig. 2A,B).

Delta power was generally shifted towards the lower values during the PW cycle compared to virgin cycle as baseline. This decrease was significant during PW MET compared to virgin MET as baseline (time X treatment, F (11, 156) = 4.31; p < 0.001) (Fig. 3A). The decreasing trend in maximal delta power seen at the beginning of the LP from MET to PRO found in case of the virgin EST cycle was less pronounced in the PW cycle (Fig. 3A).

Circadian variation of beta power increased during the PW cycle but in an antiphase fashion compared to the virgin cycle (Fig. 3D). According to this antiphase dynamics, beta power was significantly higher in the first half of all four LPs compared to the virgin cycle (time X treatment, F (47, 624) = 8.719; p < 0.001).

Gamma power values were shifted downwards during the PW cycle compared to the virgin cycle resulted in significant differences in most of the time points throughout the whole cycle (time X treatment, F (47, 624) = 2.136; p < 0.0001) (Fig. 3E).

## Discussion

This is the first study, which comprehensively reports sleep characteristics and LFP patterns throughout the reproductive cycle on the same animals. In order to separate the discussion to logical units and to compare the results with previous literature, we will first discuss sleep and LFP patterns during the estrus cycle, PREG, and the PP period under basal condition. Then, the discussion continues with the pup isolation, SDep findings and finally PW period data are interpreted.

### Estrus cycle changes

Strong REM sleep decrease during the EST night is a characteristic feature of the EST cycle and was reported several times in previous studies^9–12,14,40,41^ although one study reported no sleep changes across the EST cycle^42^ and one study reported decreased REM sleep amount even in the LP of PRO^41^ equal to the whole PRO in the present study. However, this change in REM sleep was usually named as PRO night REM sleep inhibition as all relevant sleep studies used the one day-one stage classification for the EST cycle, which method overrepresents PRO and EST and downrepresents DIE, the latter was considered to be 2 days long in case of a 4-daays EST cycle^43^. EST cycle staging used in the present study with PRO limited to the LP and EST to the DP may reflect better the time course of the level of gonadal hormones, which govern the cycle-related histological and physiological changes^26^.

REM sleep inhibition and concurrent increase of W and decrease of SWS can be explained by direct and indirect effects of E2 on cortical neurons to facilitate desynchronization and obstruct SWS initiation and/or maintenance. Indeed, E2 was found to strongly modulate inhibition in the cortex during the EST cycle by E2 β receptor-dependent mechanisms eliciting increased excitation of parvalbumin-containing fast-spiking interneurons *in vitro* and *in vivo*. Firing rate of fast-spiking interneurons was markedly lower during EST compared to MET and DIE while similar difference was not found in case of regular-spiking cells *in vivo*. Interestingly, E2 increased the level of cortical inhibition without affecting excitation^44^. Ovariectomized female rats with a high dose of E2 supplementation showed shorter SWS episodes and increased number of brief awakenings during the DP^45^.

E2 was also found to increase noradrenaline (NA) synthesis in the locus coeruleus neurons and decrease NA degradation^46^. However, at the same time, E2 reduced postsynaptic beta-adrenergic receptor functions resulting in a decreased ability of NA to affect downstream targets^47^. This results in elevated NA levels during PRO when E2 level is high but according to the drop of E2 level during EST^26^, NA level may decrease in this period of the cycle. High NA level in the cortex is crucial to maintain desynchronized state^48^ while during REM sleep, NA cells cease firing in the locus coeruleus^49^ and high NA level in the cortex inhibit the expression of REM sleep as it can be seen after the application of the α_2_-adrenoceptor autoreceptor antagonist yohimbin^50^. Taken together, high NA level evoked by high E2 release cannot be appropriate for the presence of both W and REM sleep. Another problem is the time course of the E2 level changes. High W and low REM sleep amounts can be seen even during the second part of the EST night when E2 level is significantly lower compared to the peak level seen during PRO. The contradiction can be resolved by the hypothesis that high E2 level may have a permissive role to enable cortical activation characteristic of both W and REM sleep at the end of the PRO and then high level of W and low level of REM sleep can be maintained during the EST night even if the level of E2 continues to decrease^26^. E2 has been shown to evoke behavioral activation in rats, which appears as EST behavior^51^. This behavioral activation governed by limbic structures may be an alternate source of increased W during the EST night aside from the activation, which is generated by S-W regulating structures and/or increased cortical activity evoked by E2^44^.

In addition to E2, progesterone may also have a role in the suppression of REM sleep during EST night. In ovariectomized female rats with only low-dose progesterone supplementation, no change was seen in SWS but REM sleep episodes appeared less frequently^45^.

Increased REM sleep and SWS during the LP of the MET compared to DIE2 day baseline reflected the homeostatic sleep regulation as the preceding DP was the night of the previous EST of the previous cycle with increased W as it was suggested in an earlier study^11^.

We provided here LFP analysis regarding LFP changes across the EST cycle. Compared to DIE2 baseline, beta power was the highest during PRO but elevated beta power was seen during MET LP, too. Gamma power was maximal during EST night. In a previous study^9^, LFP power between 13 and 25 Hz was found to peak during PRO night, which corresponds to EST night in the present study. Taken together, the close association of gamma activity with W^52^, increased W during EST night was reflected by the gamma- but not by the beta activity.

### Sleep and LFP during pregnancy

In the present study, sleep and LFP during PREG were analyzed on PREG10 and PREG20 days. SWS and REM sleep were decreased on the PREG10 day during the LP according to a previous study^16^, which is in line with our present results. SWS was also decreased during the DP in the present but not in previous studies^15,16^. On the PREG20 day, SWS decrease and W increase was found only in a few distinct time points without gross difference while REM sleep was suppressed both in the LP and the DP. Previous studies reported reduced diurnal SWS and REM sleep with increased nocturnal SWS and unchanged REM sleep in the same period^16^ or increased SWS only in the DP without REM sleep change neither in the LP nor in the DP^15^.

The hormonal background strongly changes during the course of PREG. PRL shows strong circadian fluctuations in the first 10 days of PREG then very low level can be seen until the 20th day and steep increase appears on the 21th and 22th days^29^. Indeed, some authors suggested an important role of PRL in the induction of PREG-associated sleep changes^10,16^. However, we showed decreased SWS and REM sleep during the PREG10 day compared to DIE2 baseline even the daily surge of PRL is expected to be present in both cases. If PRL or placental lactogens played dominant roles, sleep would be different on PREG20 day compared to DIE2 baseline due to lack of the hormone at that time period of PREG. However, SWS was similar in case of both DIE2 baseline and PREG20 day. REM sleep was reduced on PREG20 day but also REM reduction was seen during PREG10 day compared to DIE2 baseline. Intracerebroventricular or subcutaneous PRL administration failed to elicit SWS changes when applied during the LP with high sleep drive or during the LP with low sleep drive in male rats. PRL was suggested to play a role in REM sleep regulation. PRL injections had a bidirectional effect on REM sleep as REM sleep decreased in case of LP injections while increased after DP application in male rats^53^. Similar studies regarding PRL sleep effects in females have not been performed to date. In the present study, REM sleep was attenuated during the LP on PREG10 day when PRL level is supposed to be high. However, REM sleep was decreased during both phases on PREG20 day when PRL level was found to be low^29^. Overall, based on literature data, PRL does not seem to be responsible for PREG-associated SWS and W changes in the present model, but may have a role in the decreased REM sleep seen during the LP of the PREG10 day.

Decrease of REM sleep during both PREG10 and PREG20 days may be interpreted as an effect evoked by - at least partly - E2 and P. Release of both hormones was reported to increase toward the end of the PREG^29^ and both of them were suggested to suppress REM sleep^45,54^. In the present study, REM suppression was stronger on PREG20 day when hormone levels were reported to be higher compared to the PREG10 day^29^.

Elevated beta power was found during the LP at both PREG10 and PREG20 days. Beta power was increased during W on both days and also increased during SWS on PREG20 day. W beta power was similar in the LP on both days even if the amount of W was lower on PREG20 day. The latter day showed persistent beta elevation in the DP too. Taken together, arousal level during W became higher toward the end of the PREG even if the absolute W time is lower than in the first half of the PREG. Gamma power increase observed on PREG20 day indicates the same, but primarily for the nocturnal W.

Discomfort evoked by *in utero* movements of the fetus was suggested to affect sleep during PREG both in women^55^ and in rats^15^. However, deteriorative effect of fetal movement on the sleep of rat dam should be more pronounced at the second part of the PREG due to the larger size and increasing movement of the fetus. In the present study, the opposite was found as less SWS appeared on PREG10 day when the fetus size is smaller compared to PREG20 day, which showed little SWS deviation compared to DIE2 baseline suggesting that fetal movements may not increase maternal W.

### Sleep in the postpartum period

In the present study, a characteristic increase was found in W in the LP of early PP period in agreement with previous studies^15,17^. Based on the LFP data, not only the amount, but also the level of cortical arousal during W was elevated as shown by the increased beta- and gamma power. Beta activity was suggested to reflect increased sensory processing during active W^56^ while gamma activity reflects attention and behavioral activation^52^. Gamma power shows reciprocal connection with delta activity^52^, which was evident in our data as gamma elevation was associated with low delta activity during the LP.

Previous studies also reported increased W and decreased sleep during the LP in case of early PP period^15,17^. Circadian variation of sleep and W disappeared in agreement with a previous report^15^. LP SWS loss was not compensated in the DP by increased SWS time. Instead, delta power increased during the DP indicating more intense sleep^9^ suggesting intact short-term homeostatic sleep regulatory processes. Intense pup caring by the mother and the various sensory stimuli provided by the pups to their dam can both be sleep-depriving factors^15,17^. Lactating dams show prolonged kyphosis and hover over body postures for nursing their litter^57^, and these uncomfortable postures may also obstruct the induction of SWS.

According to previous studies^15,17^, decreased SWS and increased W during the LP is characteristic of the whole PP period. However, we found a different and more complex pattern. On PP9 day, W decreased below the baseline level during the whole day with simultaneously increased SWS. This may reflect long-term homeostatic sleep regulation as sleep debt accumulated during the early PP. During late PP, sleep changes disappeared and only minor changes were seen compared to DIE2 baseline. Thus, our results demonstrate that from the S-W perspective, PP period is not uniform and decreased sleep seen during the early PP period is compensated both by short- and long-term mechanisms resulting in complete sleep restoration with some rebound already on the PP9 day. After this time point, sleep seems to be consolidated as it becomes similar to the baseline level.

PP period has a complex endocrine and neurochemical background with a variety of plastic changes^31,32,58^. Prominent release of PRL and oxytocin characterize the PP period. Endogenous brain oxytocin was suggested to promote sleep but high oxytocin level induced by intracerebroventricular injection of the neuropeptide decreased both SWS and REM sleep in male rats^59^ arguing against a role of oxytocin in the changes found in the present study. However, similar studies have not been performed on females yet. Lactational hyperthermia is a prominent feature of the PP period^60–62^. Medial preoptic area plays a key role in thermoregulation^63^, maternal behavior^31,33^ and also important in sleep regulation^64,65^. Thus, medial preoptic area as an integrative center, which may be responsible for sleep changes seen during the PP period aside from the traditional ascending activating systems regulating cortical activation. Unfortunately, the lack of specific experiments manipulating the medial preoptic area and/or the ascending activating systems in mother rats leaves this question open.

### Effect of pups and lactation on the sleep of rat dams

Mother-pup separation sessions were performed to examine the effect of the pups’ presence on the sleep of the mother. In the early PP period, maternal W decreased significantly during the DP and not-significantly (but showing a trend) during the LP. This suggests that increased W seen during early PP period can be attributed – at least, partly - to the pup-derived sensory stimuli towards the mother. However, pup separation caused opposite changes during the middle PP when W was enhanced in the absence of the litter. Generally, the daily amount of W and SWS was similar during the early and middle PP separation sessions but corresponding baseline values showed remarkable difference (more W and less SWS during PP2 day compared to PP9 day). No changes were detected either in W or SWS during the late PP separation performed on PP17 day in agreement with the general view that maternal motivation is already reduced in that period as older pups are less attractive to dams^66^. It should also be considered that removal of the pups not only eliminates pup-originated inputs to the mother but PRL secretion is also ceased in the absence of suckling stimuli^18,67^. However, PRL application failed to induce changes in W or SWS in a previous study examining male rats suggesting its limited actions^53^. Therefore, it is possible that PRL has different actions in males and females. Taken these data together, pups affect the sleep of their mother in the PP period directly by external stimuli, which maybe modulated by hormones, such as PRL whose level in turn also depends on the presence of pups.

### Homeostatic sleep regulation during the EST cycle, pregnancy and PP period

In the present study, 4-h SDep sessions were performed to evaluate the possible changes of homeostatic sleep regulation during the virgin-pregnant-mother continuum. Although 6 hours is the standard duration for SDep studies, according to our previous experiences using 6-h SDep^68,69^, it is problematic to inhibit the appearance of short sleep episodes using the gentle handling method in the 5th and 6th hours without significantly stressing the animals. The 4-h gentle handling SDep was used previously in excellent relevant studies^70,71^ and data provided in the present study also indicate that even a relatively short SDep session is suitable to evaluate the changes in the homeostatic regulation in the different female reproductive stages.

Previous studies using selective REM sleep deprivation^20^, REM sleep restriction^72^, total sleep restriction^19^ or total sleep deprivation^73,74^ during the PREG focused on the consequences of maternal sleep loss regarding the different aspects of the development of the offspring. Our SDep data provide novel evidences about the dynamic changes of homeostatic sleep need during different female stages. Only one previous study performed SDep in virgin female rats to examine possible differences of the homeostatic sleep regulation across the EST cycle but found no difference between the effects of SDep sessions performed during the various stages of the cycle^9^. Based on this study, we performed SDep in virgin rats irrespective of EST cycle stage aiming only to yield a reference for comparison with PREG and PP period SDep sessions. From the 4 rats involved in the EST cycle for total SDep, 3 rats were in MET and 1 was in PRO at the morning of the SDep day. No difference was seen among the data of the individual rats (data not shown) and the data were pooled. During the PREG12 day, homeostatic regulation of SWS was found to be similar to that seen during the virgin EST cycle. However, during the PP period, homeostatic sleep need was found to be reduced, but not uniformly during the different stages of the period. Early PP period was characterized by lower SWS need during the LP, which was reflected by the finding that lost sleep cannot be recovered during the remaining 8-h long part of the LP. However, sleep debt seen at the end of the LP could be eliminated during the course of the DP. During the middle PP period, spontaneous SWS was enhanced during the whole day. However, sleep loss during the 4-h SDep could not recover until the end of the LP. Even until the end of the DP, a similar amount of sleep loss was seen both at the end of the LP and at the end of the DP indicating the lack of sleep recovery during the DP. During the late PP period SDep, the relative sleep debt compared to the amount of baseline sleep was smaller at the end of the LP than in case of the middle PP period SDep. DP SWS compensation was also missing here.

In opposition to the SWS, REM sleep loss was completely compensated by the end of the LP regardless of the stage when sleep was deprived. This finding suggests that homeostatic REM sleep regulation did not change during the EST cycle, PREG and PP period. Thus, conservation of the amount of REM sleep corresponding is a primary aim of the homeostatic sleep regulation in female rats, which can only be adjusted to the actual need in the short term even during the reproductive cycle.

### Sleep and LFP during PW compared to the virgin EST cycle

We found increased W in the first post-weaning estrus cycle as compared to the last virgin estrus cycle, which is the first report that reproductive experience has long-term effect on sleep. This result is in contrast to a previous study when no change was reported^15^. However, as no EST cycle staging was applied in the referenced study, its results are not directly comparable with the present results. LFP power analysis also showed differences between the two cycles, especially in the range of fast activities. During the PW cycle, beta power was higher during the LPs while gamma power was generally depressed compared to the virgin EST cycle. As gamma power changed in an opposite direction than beta power, a general increase or decrease of LFP power values evoked by technical reasons (for example, deterioration of electrode functions evoked by reactive gliosis due to the long-lasting experiments) during the PW cycle can be ruled out as an explanation for the observed LFP changes. Sleep and LFP changes detected between the PW and the virgin estrus cycle may be the results of the previous maternal experience and the accompanying neuronal and hormonal adaptations, which may have induced long-term neuroplastic changes affecting S-W regulation. Maternal experience was found to enhance spatial cognition even in PW^75^ indicating the existence of maternal experience-dependent long-term plasticity in the hippocampus possibly due to epigenetic alterations^76^ but similar studies regarding S-W regulating structures have not been performed yet. Alternatively, these changes may represent only the first PW estrus cycle, or the normal age-dependent change of the EST cycles independently from the maternal experience. It would be interesting to test the latter possibility in age-matched virgin rats as in the recent study at least 8 weeks elapsed between the onset of the virgin and PW cycles.

### General view of the maternal sleep

PREG represents a crucial point in female sleep regulation. As time progresses, needs of the fetuses and later of the pups may increasingly dominate the actual needs of the mother. The wide variety and high number of subjective and objective problems regarding sleep during PREG and the PP period in women^3,77,78^ may reflect this conflict between the intrinsic sleep needs of the mother and the actual sleep, which is allowed to be expressed in the presence of the fetuses or pups. From this point-of-view, sleep and LFP changes during PREG and the PP period reported here can have functional significance. Fetuses and pups need the W, SWS and REM sleep of their mother but the actual need in a given time point or period for a given vigilance level may change. W of the mother is needed to provide nutrition and sensory stimulation to the fetuses and later to the pups for proper intrauterin and extrauterin development^38,39^. During the PP period, W is also crucial for the expression of different maternal behaviors including intense licking of the pups to elicit urination and defecation, which is crucial for the pups’ survival^79^. Maternal REM sleep during PREG is important for the development and maturation of S-W-related neuronal networks of the pups^20^. REM sleep restriction during PREG resulted in impaired ultrasonic vocalization of the pups indicating developmental cognitive deficits^72^ and an elevation in the hippocampal level of brain derived neurotrophic factor in the pups^19^, which may result in improper formation of synaptic connectivity and plasticity^80^.

Importance of maternal SWS was examined only in two previous studies. Total sleep deprivation for 5-h during the third trimester resulted hyperactivity and increased risk-taking behavior of the offspring^73^. 6-h total SDep applied for 7 consecutive days during PREG caused impaired hippocampal-dependent spatial learning and memory, increased depressive- and anxiety-like behaviors and reduced adult hippocampal neurogenesis in the offspring^74^. However, as REM sleep was also deprived in these studies, it is hard to assume the role of SWS deprivation itself in the reported changes.

Results of SDep in the present study supports this view. Independently from the female/maternal period when it was applied, REM sleep was completely recovered or even a positive balance was seen for the REM sleep at the end of the day. This indicates that maternal REM sleep may bear tremendous importance also for the fetuses and later for the pups. However, this is not the case for SWS as it failed to recover in the middle and late PP period even by the end of the whole day. This suggests that maternal SWS may bear less importance regarding the pups and strong rebound after the SDep in the PP period may obstruct the appropriate amount of maternal care, which is related to W. Changes in the maternal homeostatic sleep regulation may also serve the maternal care. Reduced homeostatic sleep drive may enable more W, which is needed for the expression of maternal behavior. However, lactation may limit the decrease of the spontaneous maternal SWS below a limit as SWS epochs are prerequisites for reflex milk ejection^81^. Conversely, lactation was suggested to play a role in SWS provocation in rat dams as suckling stimuli increased plasma PRL level resulting in the induction of SWS^18,21^.

## Methods

### Surgical procedure and housing

Experiments were performed on 10 female Wistar rats aged 10–11 weeks and weighing between 240 and 290 g at the time of the surgery. Electrode implantation was carried out under i.p. ketamine (80 mg/kg)/xylazine (10 mg/kg) anesthesia. Rats were placed into a stereotaxic frame (David-Kopf) then the skin on the head was opened in the midline and muscles were retracted. To record LFP activity, bipolar concentric electrodes (125 µm polyimide insulated stainless steel wire fixed in a 23 G stainless steel tube) were implanted at the vertex (Br: −4, 5, L: 2.0) on both sides. The tube touched the cortical surface, while the wire reached layer VI 1.5 mm below. 1.1 mm stainless steel screw (Fine Science Tools) was implanted into the bone over the cerebellum was used as reference and ground. To monitor EMG activity, a pair of Teflon-insulated stainless steel wire of 250 μm of diameter each was inserted into the neck musculature. All leads were soldered to a miniature connector prepared from a standard 50 × 2 connector strip (Precision) and the skull was covered with acrylic resin (Cranioplastic cement, Plastic One). Recording sessions started after 1–2 weeks of recovery. The surgery and electrode implantation was similar to that published earlier by our laboratory^1^. Following surgery, the animals were kept warm, and painkillers (50 mg/kg metamizole, i.p.) was administered for 3 days.

Rats were kept in a LD12:12 cycle (lights on at 10:00 h) and were housed in individual cages located in a sound-attenuated room throughout the whole experiment. The cages were prepared from clear Plexiglas cylinders (height: 330 mm, diameter: 300 mm). Water and standard laboratory chow were available *ad libitum*.

Rats were connected to the recording apparatus two days before the first recording session to allow habituation to the recording situation. Flexible flat cables connected the rats to swivels fixed above the large Plexiglas cylinders during the recordings. Cables were folded to a zigzag shape with a rubber string running in the middle to provide free movements of the rat. The experimental setup was similar to that published earlier by our laboratory^1–3^ and others^4^.

After the verification of the PREG, paper nest material was placed to the cages to facilitate nest building. The number of pups was adjusted to 7–8 within 2 days of parturition. All recordings and treatments were done in the home cages. Pups were housed together with their dam during the whole PP period except the 24-h mother-pup separation sessions. During the separation, pups from the same litter were housed together in the same room as their dam, but in a far distant corner. The number of pups raised by the rats was 7.3 ± 0.7.

Experiments were carried out in accordance with the Hungarian Act of Animal Care and Experimentation (1998, XXVIII) and with the directive 2010/63/EU of the European Parliament and of the Council of 22 September 2010 on the protection of animals used for scientific purposes. Experimental protocols were approved by the Ethical Board of Eötvös Loránd University. Efforts were made to minimize the number of animals used.

### Experimental timeline and baseline definition

#### EST cycles

Virgin EST cycle and PW EST cycle was characterized by 120-h long recordings running without interruption. For the analysis, DIE2 day was taken as baseline for virgin EST cycle. PW recordings started after 5 days of the offspring removal. In this case, the whole virgin EST cycle was taken as baseline for point-to-point comparison.

#### Pregnancy

First and second half of PREG was characterized by representative 24-h recordings made on PREG day 10 (PREG10) and 20 (PREG20), respectively. PREG10 and PREG20 values was compared with DIE2 baseline values.

### Postpartum baseline

Early, middle and late PP was characterized by recordings made on PP days 2 (PP2), 9 (PP9) and 16 (PP16), respectively. DIE2 was taken as baseline for comparison. PP period was limited to 21 days then offspring were removed from their mother on the next morning and housed separately.

### Postpartum treatment – mother-pup separation

Mother-pup separation sessions were conducted on PP days 3 (PP3), 10 (PP10) and 17 (PP17). Corresponding PP baseline recordings served as baseline (PP2 for PP3 separation; PP9 for PP10 separation; PP16 for PP17 separation). Averaged baseline values were calculated from the identical rats subjected to the separation session.

### Total sleep deprivations

All total SDep session started at lights on, lasted for 4-h and performed by the gentle handling method. After the deprivation period, recordings were continued for further 20 hours without interruption. SDep data were compared to averaged baseline values calculated from the identical rats subjected to the actual SDep session.

### EST cycle SDep

4-h SDep sessions started at the LP onset. SDep was performed 2 days after the end of the 120-h first recording session on virgin rats, irrespectively on the EST cycle stage of individual rats. EST cycle phase was determined by vaginal smear cytology at the morning of the SDep session, but data were pooled later. SDep session preceded the first mating session. DIE2 was taken as baseline for comparison.

### Pregnancy SDep

SDep was done on PREG12 day during the second half of the PREG. PREG10 day was defined as baseline. As PREG20 day was distant in time, PREG10 day was selected because of the smaller time difference between PREG12 and PREG10 days.

### PP period SDeps

SDep sessions were performed on PP4, PP11 and PP17 days, representing the early, middle and late PP period, respectively. Corresponding baseline recordings were PP2 for PP4, PP9 for PP11 and PP16 for PP17.

All recording session started at light on (10:00 AM) and lasted for 24-h or 120-h without interruption. Overview of the experimental timeline is illustrated by Fig. 1. Note that not all rats involved in the experimental schedule received all treatments/manipulations.

All treatments (mother-pup separation, SDep) were performed using a randomized fashion with one restriction. Effort was made to limit the number of separation sessions for maximum two in case of a given dam and their pups to avoid the possible deterioration effect of fasting on pups’ development. At least two days elapsed between two consecutive tretaments to avoid the interference between the sleep effects of the manipulations.

### Vaginal smear cytology

Vaginal smear sampling was performed at the light onset on the first day of the 120-h recordings and at the next light onset after the end of the 5-day recording. Sampling was not performed daily during the uninterrupted recordings to avoid the non-specific sleep effects of the sampling procedure. Vaginal smear samples were dried to glass slides and stained by Giemsa solution. Stages of the EST cycle were identified according to standard histological criteria^82^. MET was characterized by cornified epithelial cells and leukocytes. DIE smear contained nucleated epithelial cells, leukocytes, mucus and cellular debris. PRO smear was consisted of mainly nucleated epithelial cells while EST smear was dominated by anucleated cornified epithelial cells.

### Estrus cycle staging, mating and pregnancy verification

Length of the EST cycle was defined as 96 hours. MET duration was taken to be 24 hours, DIE 48 hours (1st day – DIE1; 2nd day – DIE2) while PRO and EST 12–12 hours, respectively, according to the conventional literature values^43^. According to the hormone release profiles, PRO occurs during the LP while EST mainly involves a single DP in case of the generally used LD12:12 lighting conditions^26,28^. DIE2 was taken as baseline/reference for virgin EST cycle, PREG and PP baseline recordings.

EST cycle staging was performed using three different way of information. First, vaginal smear cytology, second, the date of the verified PREG and third, the very characteristic REM sleep reduction seen during one DP, or rarely, on two DPs in the 120-h recordings involving five DPs. EST night REM sleep reduction was seen in case of each rats during the virgin- as well as during the PW cycles. As PREG onset appears during EST and in most cases only 3–4 days elapsed between the end of the 120-h EST cycle recording and the onset of the PREG, the latter had helped to verify the result of vaginal cytology performed the next morning after the recording was completed.

For the mating sessions, female rats were disconnected from the recording system and housed together with a male rat from the same strain for 24 hours then PREG was checked. In case of negative result, mating sessions were repeated until PREG occurred. The presence of spermatozoids in the vaginal smear at the following morning was considered to be a positive indicator of PREG, according to the standard criteria^43^. After the verification of the PREG, female rats were reconnected to the recording cables and electrophysiological recording continued. Average duration of PREG was 22 ± 0.58 days.

### LFP recording and sleep deprivation

LFPs were recorded between the vertex wire electrodes inserted to layer 5 and cerebellar reference screw through home-designed headstages based on TLC2264I (Texas Instruments, USA) operational amplifiers built into the male connector. Signals from the both sides were recorded, but the best signal with least artifacts was analyzed.

Signals were amplified (500x), filtered (0.3 Hz–5 kHz) and sampled with 8192 Hz then digitalized at 16-bit resolution by an analog-to-digital (A/D) converter card (Labview, National Instruments, Austin, TX, USA) and stored by a custom-made software for further analysis. Narrow frequency range for filtering and high sampling rate was used to record layer 6 multiple unit activity besides the LFP, but multiple unit data were not analyzed in this study. Recorded data was downsampled to 128 Hz offline to yield 512 point (a power of 2) in 4 s in order to facilitate Fast Fourier Transformation (FFT) of the signals. EMG was filtered in the same way for technical reasons. All LFP and EMG data obtained during the recording sessions were stored on hard disk for off-line analysis.

Total SDep was induced by gentle handling of the animals for 4 hours starting at the lights-on period. Gentle handling was performed in the home cages and involved presentation of new objects, acoustic and if necessary, tactile stimulation to the rats^83^.

## Data analysis

### Sleep scoring and analysis of rebound sleep

Sleep stages were scored using custom-made semi-automatic software. The program enabled visual inspection of the recorded signals, digital filtering, and spectral analysis of the LFP curves. Power spectra were calculated using the FFT algorithm for all consecutive 4-s periods from all recordings. Power was integrated in the delta (0.5–4 Hz), theta (4–10 Hz), alpha/sigma (10–16 Hz), beta (14–30 Hz) and gamma (30–48 Hz) frequency ranges and the ratio of the theta/delta power determined. EMG data was also processed using the FFT method and the total power (variance) was calculated in the 5–48 Hz range.

Epochs containing movement artifacts (high delta power and high EMG variance) and rapid eye movement (REM) sleep epochs (low delta power, high theta/delta ratio, and low muscle tone) were manually selected in all recordings by visual inspection of the LFP and EMG signals^5–7^.

Epochs containing artifacts were excluded from further analysis (less than 1% of the baseline recording and recovery and around 5% during SDep).

In the present experiments, delta power and EMG thresholds were set individually for each rat by visually inspecting of the raw LFP and EMG data from control recordings. These objective thresholds were then used to score recordings obtained after the treatments. Epochs in which delta power was above and EMG value below these thresholds, respectively, were marked as SWS, while epochs with lower delta power or higher EMG activity as W. Scoring was performed by a semi-automated method had been published earlier^84^ and was used in several studies published by our laboratory later^50,68,69,85–87^. Raw hypnograms were smoothed, i.e. every 32-s period was assigned to the dominant S-W stage^88^.

To examine the effect of the total SDep on sleep and the following rebound, amounts of SWS and REM sleep were analyzed in four different time periods. Delta power was also calculated in the same four periods. As rebound sleep was expected to be the most pronounced in the first two hours after the end of the SDep, these hours (5^th^ and 6^th^) were analyzed separately. In some cases, the first, intense rebound seen in the 5^th^ and/or in the 6^th^ hour was not able to completely compensate for the lost sleep and delta power. To assess long-term recovery processes, S-W stages and delta power changes were followed to the end of the LP, and until the end of the DP.

### LFP power analysis

LFP power values were analyzed by both independently and dependently from the vigilance stages^52,89–91^. Power values in the delta (1–4 Hz), theta (4–10 Hz), alpha/sigma (10–16 Hz), beta (16–30 Hz) and gamma (30–48 Hz) frequency bands independently from the vigilance levels (W, SWS, REM sleep) were averaged for 60-minute long periods and normalized using the data of the defined baseline day (DIE2 day of the virgin EST cycle) which was used as reference for all other recordings regardless of their type (other baseline for a separate stage etc.). Grand average of the power values of all hours and frequency bands of the baseline day was calculated as’normalization factor’. Then each of the power values belonging to any time point were divided by the same’normalization factor’ in case of both the baseline and the treatment recordings. Normalized values then summarized for 120-minutes long periods. After that, values of the 2-h periods of the baseline day were averaged separately in the different bands. Actual values of the 2-h periods were divided by this daily average and expressed as average % by frequency bands. Normalized values belonging to the treatment days were also divided by the baseline daily average separately for each frequency bands and expressed as baseline %.

Vigilance stage dependent LFP analysis was performed when the independent analysis showed significant power change in a given frequency band in one or more time points to assign the change of the power to one or more S-W stages. For this, LFP power values in all five frequency bands were computed by vigilance levels. Then power values were normalized separately for all three vigilance levels using a separate averaged power (’normalization factor’) value for each of the vigilance levels. Normalizing values were derived from the baseline (DIE2 day of the virgin EST cycle) recording. Then normalized values belonging to a given frequency band were compared between baseline versus treatment days separately for W, SWS and REM sleep. Aside from the comparisons between baseline and treatments, this kind of analysis also demonstrated the inherent differences in the distribution of power values of a given frequency band between the different S-W stages.

Data from the 0–1 Hz LFP range were completely excluded from the analysis as artifacts originating from cable movements fell mostly in this range.

### Statistical analysis

S-W parameters (time, epoch number and epoch length) and normalized LFP power values expressed in baseline% were analyzed statistically by two-way ANOVA with time and treatment as factors, followed by Sidak’s multiple comparisons test. Sidak’s test has no assumption that the number of samples in each group is the same. This post-hoc test is theoretically valid with independent, uncorrelated outcomes only. Althought Sidak’s test is liberal in case of negatively correlated data, it is conservative when the correlation is positive. As the outcome of the correlation between sleep datasets can be both, this can be evaluated as some kind of balance. In case of the SDep analysis, SWS and REM sleep values were also summarized for two long periods (5–12 hours of the LP; 5–24 hours containing LP after the SDep and the DP), respectively, in case of the SDep day and the baseline day, respectively. In this case, sleep data were compared by Welch’s t-test. In each series of experiments, homogeneity of variances and normal distribution of data was tested before statistical analysis. All tests were two-tailed and p < 0.05 was accepted as the lowest limit of significant difference. Data are shown as mean ± S.E.M. in figures. The term „timeXtreatment” reflects the interaction between the factors indicated by the two-way ANOVA. Statistical analysis was performed using Prism 7.0 (GraphPad Software, San Diego, USA). Data were plotted in Microcal Origin 8.0 (OriginLab Corporation, Northampton, USA). Final editing was performed using Adobe Photoshop CC.

## Supplementary information

Supplementary Information.

## Data Availability

All data analyzed and presented in this work are available from the corresponding author upon reasonable request.
