# Supplementary material for: Complete sleep and local field potential analysis regarding estrus cycle, pregnancy, postpartum and post-weaning periods and homeostatic sleep regulation in female rats
Source: Sci Rep. 2020 May 22;10:8546. doi: 10.1038/s41598-020-64881-w (PMC7244504; doi:10.1038/s41598-020-64881-w)
Supplement: Supplementary file 1 — Supplementary Information. [file 41598_2020_64881_MOESM1_ESM.docx]

**Complete sleep and local field potential analysis regarding estrus cycle, pregnancy, postpartum and post-weaning periods and homeostatic sleep regulation in female rats**

Attila Toth^1^; Mate Petho^1^, Dora Keseru^1^, Dorina Simon^2^, Tunde Hajnik^1^; Laszlo Detari^1^, Arpad Dobolyi^2^

Corresponding author:

Attila Toth, PhD^[[1]](#footnote-1)^

^1^*In vivo* Electrophysiology Research Group, Department of Physiology and Neurobiology, Eötvös Loránd University

^2^MTA‑ELTE Laboratory of Molecular and Systems Neurobiology, Department of Physiology and Neurobiology, Hungarian Academy of Sciences and Eötvös Loránd University

**Supplementary Figures**

**
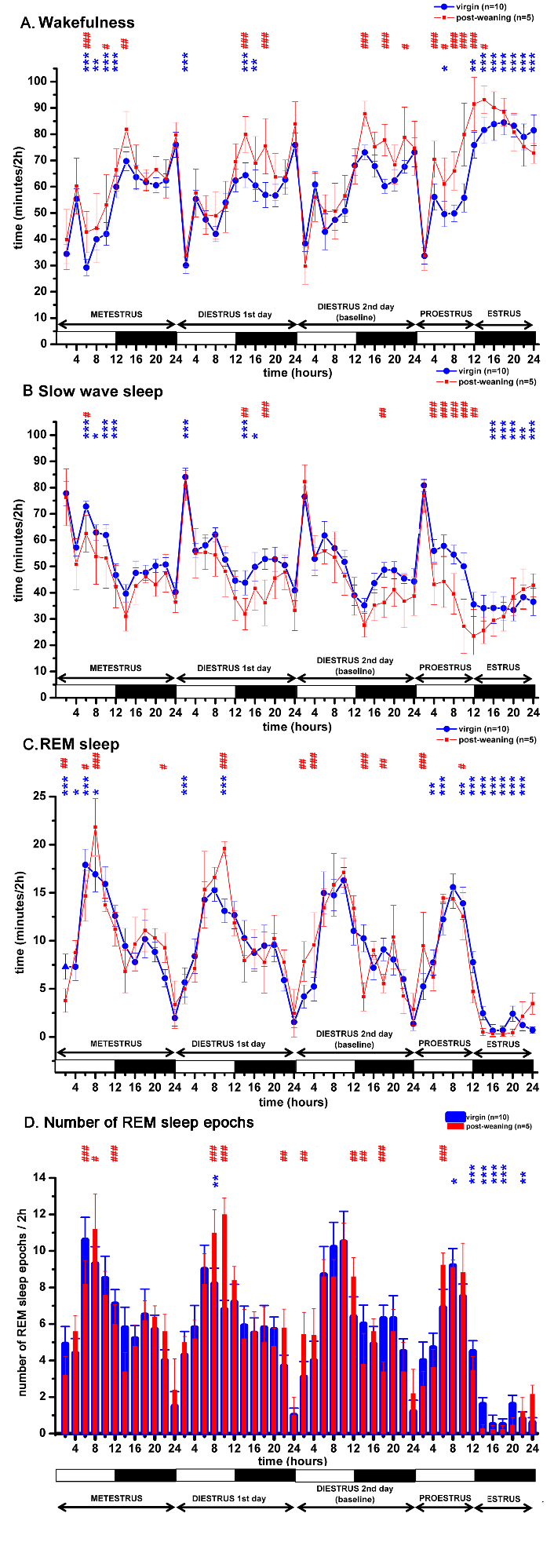
**

**Figure S1**. S-W changes during the virgin EST cycle (n=10, thick blue line) and during the PW EST cycle (n=5, thin red line). Panel A: W; panel B: SWS; panel C: REM sleep; panel D: number of REM sleep epochs. Data were analyzed in 2-h long bins and expressed as minutes/2-h. MET duration was taken to be 24 hours, DIE 48 hours (DIE 1st day and DIE 2nd day) while PRO and EST 12-12 hours, respectively. White and black bars at the X axis represent light- and DPs, respectively. Blue asterisks (*) indicate significant deviation from the corresponding baseline (DIE2 day) value in case of the virgin EST cycle data. Red hashes (#) indicate significant deviation in a point-to-point comparison from the corresponding reference (virgin EST cycle) value in case of the PW EST cycle data.

Significance was tested with two-way ANOVA with time and treatment as factors, followed by Sidak's multiple comparisons test. Significance levels: *,# - p < 0.05; **,## - p< 0.01; ***,### - p < 0.001. Data are expressed as mean ± S.E.M.

**
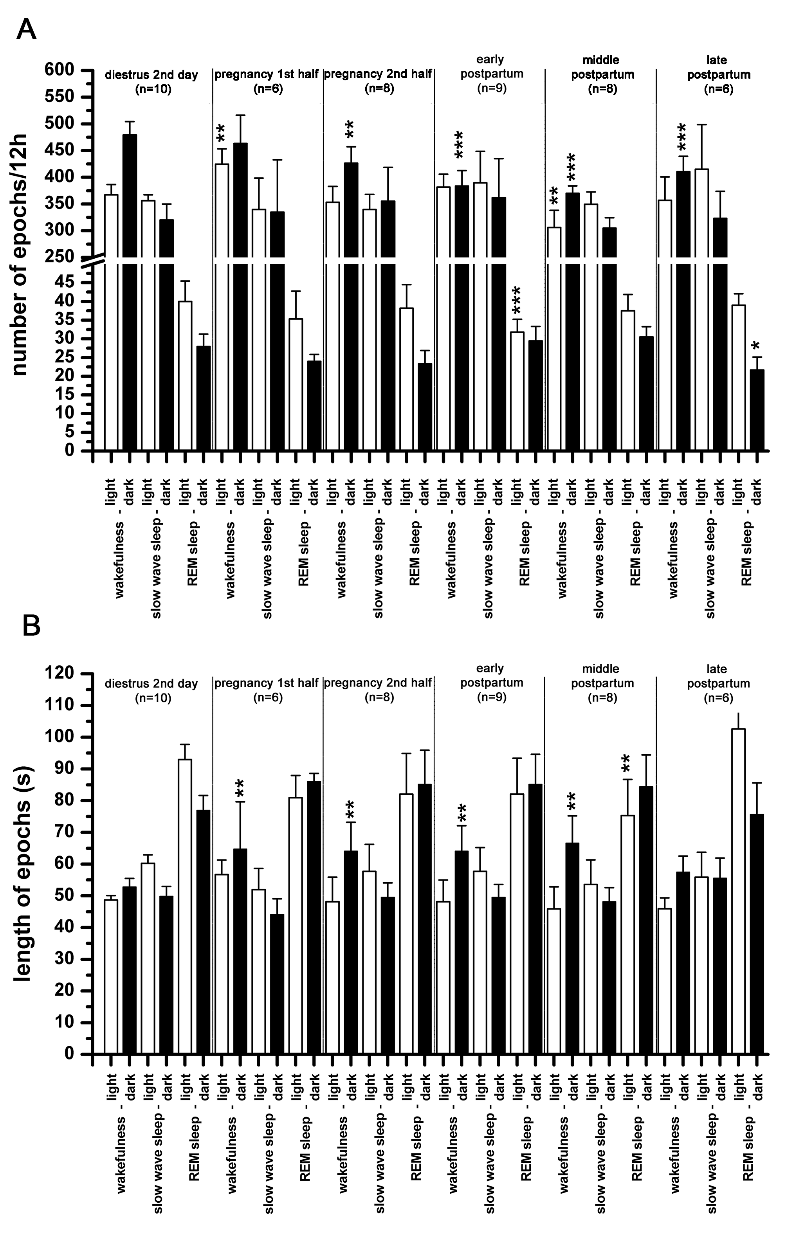
**

**Figure S2**. Averaged number (panel A) and averaged length (in seconds; panel B) of S-W epochs of the 12-12 hours light-DPs during the DIE2 day of the virgin EST cycle (n=10), during the first half of the PREG (PREG10 day, n=6), second half of the PREG (PREG20 day, n=8), early PP (PP2 day, n=9), middle PP (PP9 day) and late PP (PP16 day, n=6). Black asterisks (*) indicate significant difference compared to the LP and DP values, respectively, of DIE2 as reference, respectively.

Significance was tested with two-way ANOVA with time and treatment as factors, followed by Sidak's multiple comparisons test. Significance levels: * - p < 0.05; ** - p< 0.01; *** - p < 0.001. Data are expressed as mean ± S.E.M.

**
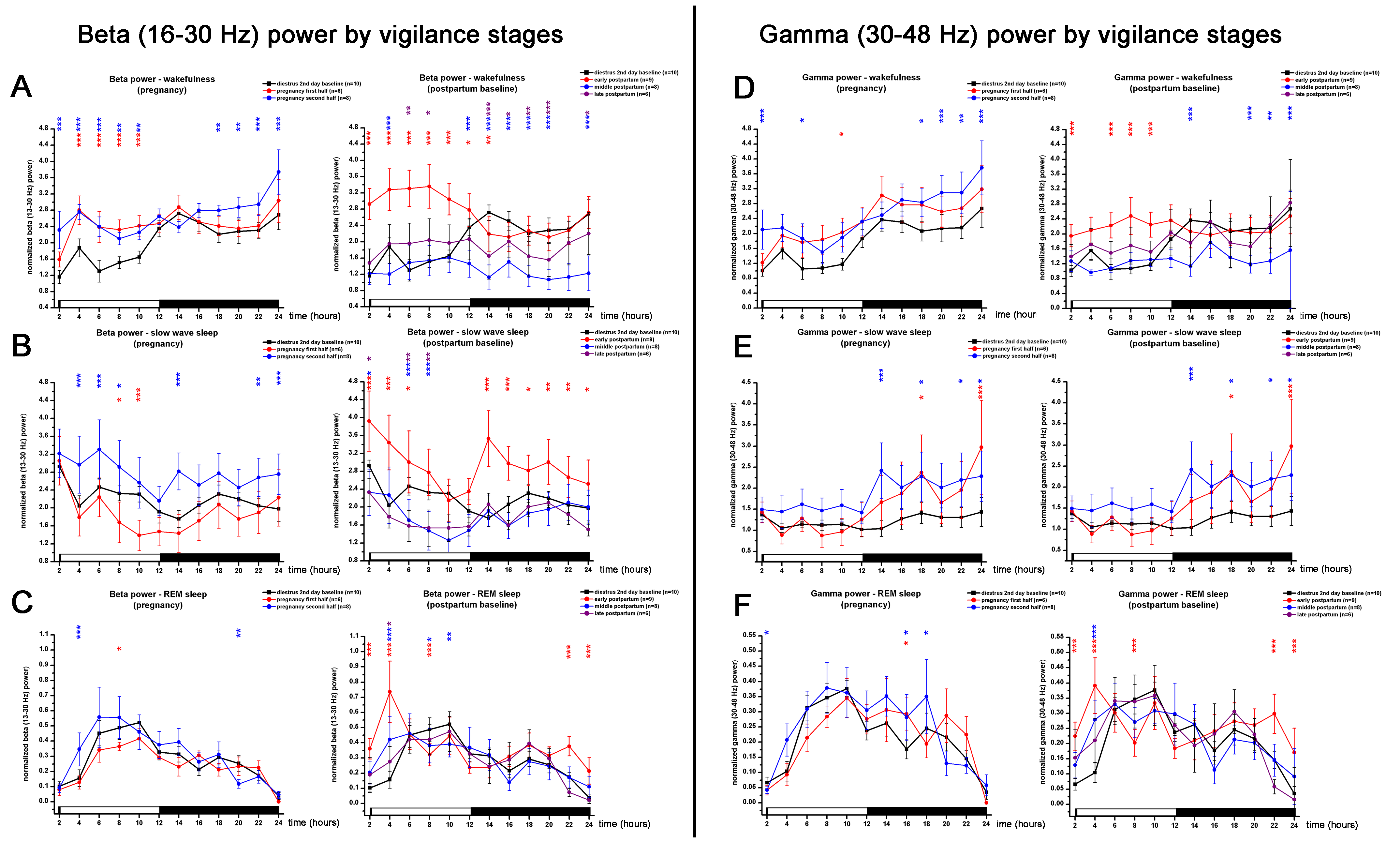
**

**Figure S3**. LFP beta (left, 16-30 Hz) and gamma (right, 30-48 Hz) power by vigilance levels (beta power - panel A: W, panel B: SWS, panel C: REM sleep; gamma power - panel D: W, panel E: SWS, panel F: REM sleep) during the DIE2 day of the virgin EST cycle (n=10), during the first half of the PREG (PREG10 day, n=6), second half of the PREG (PREG20 day, n=8), early PP (PP2 day, n=9), middle PP (PP9 day) and late PP (PP16 day, n=6). Power spectras were normalized separately for each vigilance levels and normalized power values were compared using DIE2 day of the virgin EST cycle as baseline. Only beta and gamma power values were depicted here. White and black bars at the X axis represent light- and DPs, respectively.

Red asterisks (*) indicate significant deviation during PREG10 day (left column) or PP2 day (right column), respectively, compared to DIE2 baseline. Blue asterisks (*) indicate significant deviation during PREG20 day (left column) or PP9 day (right column), respectively, compared to DIE2 baseline. Purple asterisks (*) indicate significant deviation during PP16 day (right column), respectively, compared to DIE2 baseline.

Significance was tested with two-way ANOVA with time and treatment as factors, followed by Sidak's multiple comparisons test. Significance levels: *,*,* - p < 0.05; **,**,** - p< 0.01; ***,***,*** - p < 0.001. Data are expressed as mean ± S.E.M.

**
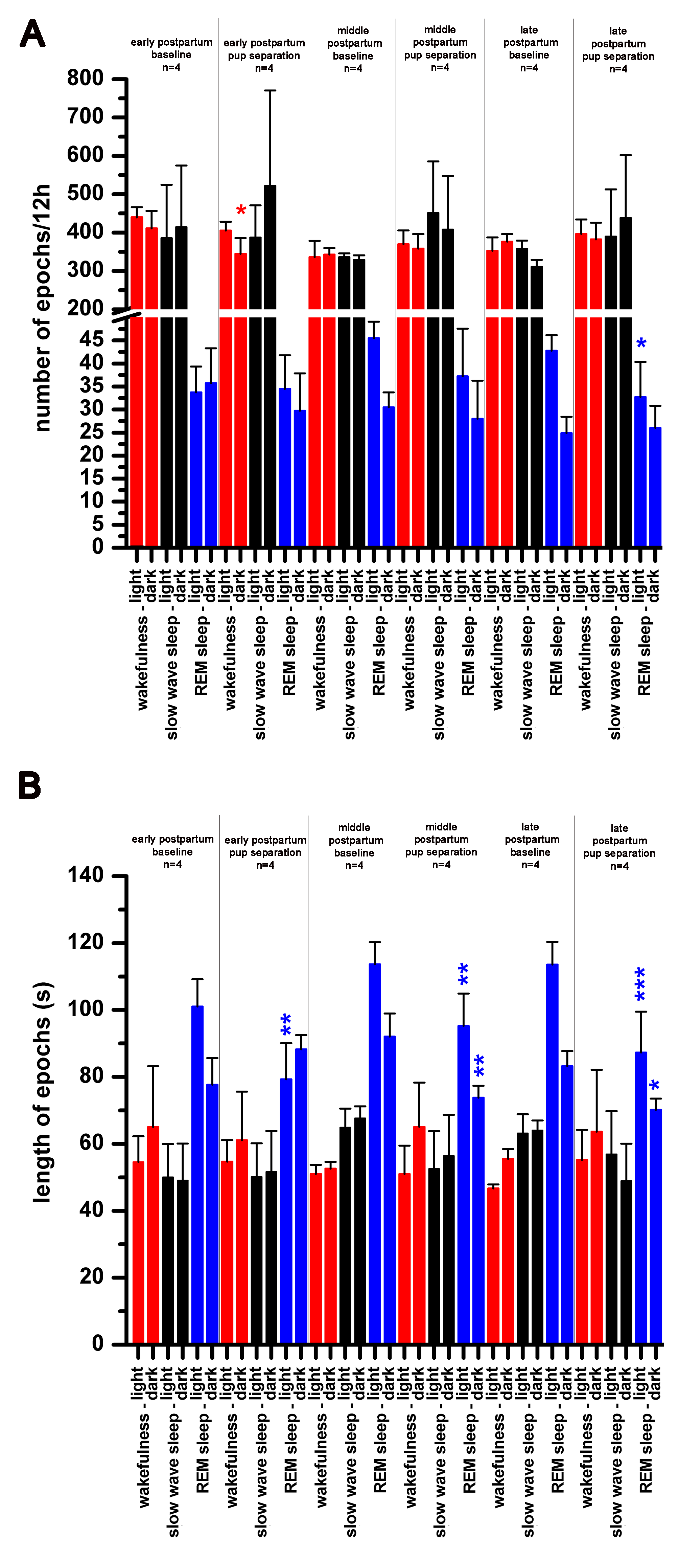
**

**Figure S4**. Averaged number (panel A) and averaged length (in seconds; panel B) of S-W epochs of the 12-12 hours light-DPs during separation sessions performed during early PP (n=4, PP3 day), middle PP (n=4, PP10 day) and late PP (n=4, PP17 day). Corresponding PP baseline recordings served as baseline (n=4, PP2 for PP3 separation; n=4, PP9 for PP10 separation; n=4, PP16 for PP17 separation; n=5). Red columns: W, black columns: SWS, blue columns: REM sleep. Averaged baseline values were calculated from the identical rats subjected to the actual treatment in each cases.

Red asterisks (*) indicate significant difference in W epoch number or length during the LP and DP, respectively, compared to the corresponding baseline. Black asterisks (*) indicate significant difference in SWS epoch number or length during the LP and DP, respectively, compared to the corresponding baseline. Blue asterisks (*) indicate significant difference in REM sleep epoch number or length during the LP and DP, respectively, compared to the corresponding baseline.

Significance was tested with two-way ANOVA with time and treatment as factors, followed by Sidak's multiple comparisons test. Significance levels: *,*,* - p < 0.05; **,**,*- - p< 0.01; ***,***,*** - p < 0.001. Data are expressed as mean ± S.E.M.

1. ^,2^ Department of Physiology and Neurobiology, Eötvös Loránd University,

   Pázmány Péter sétány 1/C, Budapest 1117, Hungary

   Tel.: +36-1-381-2181; fax: +36-1-381-2182

   e-mail: attila.toth@ttk.elte.hu [↑](#footnote-ref-1)
